# Subtropical coastal microbiome variations due to massive river runoff after a cyclonic event

**DOI:** 10.1186/s40793-024-00554-9

**Published:** 2024-01-30

**Authors:** M. Meyneng, H. Lemonnier, R. Le Gendre, G. Plougoulen, F. Antypas, D. Ansquer, J. Serghine, S. Schmitt, R. Siano

**Affiliations:** 1 IFREMER, DYNECO, BP70, Plouzané, France; 2 French Institute for Research in the Science of the Sea (IFREMER), Research Institute for Development (IRD), University of New Caledonia, University of Reunion, CNRS, UMR 9220 ENTROPIE, Nouméa, New Caledonia

**Keywords:** Microbial dynamics, Environmental DNA, Extreme events, River runoff, Land–sea continuum, New Caledonia

## Abstract

**Background:**

Coastal ecosystem variability at tropical latitudes is dependent on climatic conditions. During the wet, rainy season, extreme climatic events such as cyclones, precipitation, and winds can be intense over a short period and may have a significant impact on the entire land‒sea continuum. This study focused on the effect of river runoff across the southwest coral lagoon ecosystem of Grand Terre Island of New Caledonia (South Pacific) after a cyclonic event, which is considered a pulse disturbance at our study site. The variability of coastal microbiomes, studied by the metabarcoding of V4 18S (protists) and V4–V5 16S (bacteria) rDNA genes, after the cyclone passage was associated with key environmental parameters describing the runoff impact (salinity, organic matter proxies, terrestrial rock origin metals) and compared to community structures observed during the dry season.

**Results:**

Microbiome biodiversity patterns of the dry season were destructured because of the runoff impact, and land-origin taxa were observed in the coastal areas. After the rainy event, different daily community dynamics were observed locally, with specific microbial taxa explaining these variabilities. Plume dispersal modeling revealed the extent of low salinity areas up to the coral reef area (16 km offshore), but a rapid (< 6 days) recovery to typical steady conditions of the lagoon's hydrology was observed. Conversely, during the same time, some biological components (microbial communities, Chl *a*) and biogeochemical components (particulate nickel, terrigenous organic matter) of the ecosystem did not recover to values observed during the dry season conditions.

**Conclusion:**

The ecosystem resilience of subtropical ecosystems must be evaluated from a multidisciplinary, holistic perspective and over the long term. This allows evaluating the risk associated with a potential continued and long-term disequilibrium of the ecosystem, triggered by the change in the frequency and intensity of extreme climatic events in the era of planetary climatic changes.

**Supplementary Information:**

The online version contains supplementary material available at 10.1186/s40793-024-00554-9.

## Background

Coastal areas are among the most productive, exploited and threatened systems on earth [1]. The microbial component plays a crucial role in the functioning and sustainment of the diverse ecological and economic benefits and services offered by these ecosystems [2, 3]. Hence, spatiotemporal variations in the taxonomic and functional composition of microorganism communities can have a direct effect on coastal environment functioning. While global patterns were highlighted in the ocean microbiome by worldwide expeditions [4–7], coastal microbial dynamics show even greater variability [8, 9] on daily [10], seasonal [11, 12] or interannual scales [13, 14], and in narrow geographic areas [15]. This variability is mostly due to variation in terrigenous inputs advected along the land‒sea continuum, especially from river runoff, which is the main source of nutrients, organic matter (OM), and even pollutants [16]. River inputs are themselves subject to a certain variability [17]. Their quantity and quality depend on watershed characteristics (size, type of soil, relief, vegetation, etc.) and climatic conditions (seasonality, extreme events, etc.), but also on human activities [18]. The adoption of a holistic and cross-habitat perspective is crucial to assess the close relationship existing between the land and coastal waters to better understand coastal microbiome dynamics and their effects on the whole ecosystem.

Among the microbiome dynamics of the different worldwide oceanographic areas [19], subtropical and tropical regions show a typical pattern with relatively low variability in primary production throughout the year [20]. In these zones, primary production is strongly dependent on climate and meteorological events, which can directly change coastal ecosystem functioning at local scale [21]. In general, tropical and subtropical climates are characterized by two seasons: the rainy and dry seasons [22]. The specificity of these regions is the occurrence of extreme climatic events, with a mean of 85 storms per year observed along the tropical belt during this season [23]. Cyclones (defined as storms formed in the South Pacific or Indian Ocean) are intense and short-term disturbances generating environmental perturbations along their trajectories. At regional scale, such as on the northwest coast of Australia, tropical cyclones can contribute to 55% of seasonal rainfall (November–April) [24]. The consequences of a storm passage are the input of matter, advected organisms from terrestrial ecosystems in marine coastal ecosystems due to river runoff after soil washout, as well as an increase in nutrient concentrations and a thickening of the mixed layer in the water column, a drop in salinity and a reduction in light penetration [25–29]. Few studies have followed the evolution of the microbial compartment after a cyclonic event in the tropics and subtropics. They notably showed an increase in chlorophyll *a* [30–32], changes in taxonomic composition [33–35] and modifications of functional gene activities [36], supporting the idea of a perturbation of microbial communities at both taxonomic and functional levels. The resilience of communities and ecosystems, defined as the time required for the system to return to its post-disturbance state [37], depends on cyclone intensity, trajectory, and local hydrodynamic characteristics. The parameters considered to analyze the recovery, which represent different parts of the system, may have distinct recovery dynamics. For instance, values of chlorophyll *a*, a proxy of phytoplankton biomass, can return to the normal range after 5 days [30], but eukaryotic communities can still be disturbed after a month [38].

In the South Pacific, the coastal ecosystems of the New Caledonia archipelago are strongly influenced by cyclonic events and river runoff [39, 40]. This French overseas territory is well representative of widespread tropical ecosystems influenced by riverine inputs, such as other high islands (Fidji, Hawaï, French Polynesia, etc.) or shelf seas (Australia, Brazil, India, etc.). The main island, called the “Grande Terre”, has numerous rivers whose streams and coastal runoffs are influenced by climatic variability, such as cyclones, which can multiply the flow rate by a hundred [40]. The influence of river inputs on the biogeochemical characteristics of the southwest (SW) lagoon of Grande Terre has been shown to cause a coast-offshore biogeochemical gradient with an increase in suspended particulate matter, metal concentration, nutrients and chlorophyll *a* close to the land [41–43]. This spatial gradient is mirrored in the microbial compartment in terms of phytoplanktonic community composition and bacterial production [44–47]. In addition, a temporal pattern has been highlighted at seasonal (dry and wet seasons) [44] and interannual [48] scales in the lagoon, with changes in the microbial community. Those studies are based on pigments, cytometric and microscopic methods, but it is now strongly acknowledged that environmental genomic approaches (analyses of environmental nucleic acids) allow us to more precisely assess the structure and composition of the complex microbial diversity [49]. The fine characterization of the microbial community by environmental DNA (eDNA) coupled with analyses of environmental parameters after the passage of a cyclonic disturbance would provide new insights into coastal microbiome dynamics.

The aim of this study was to characterize the response of subtropical oligotrophic ecosystems affected by massive river runoff derived from cyclone disturbance using environmental genomic approaches. The Grande Terre of the New Caledonia archipelago was used as our study case. This analysis was assessed by (1) associating hydrological and biogeochemical variations with biological community variations (protist and bacteria); (2) studying the structuration and seasonal changes in the microbial communities during two campaigns in the dry season, which were used as baseline ecological conditions for assessing variations after a cyclonic event; and (3) evaluating microbiological community dynamics at a daily time scale after the cyclone passage.

## Methods

### Study area

**Fig. 1:**
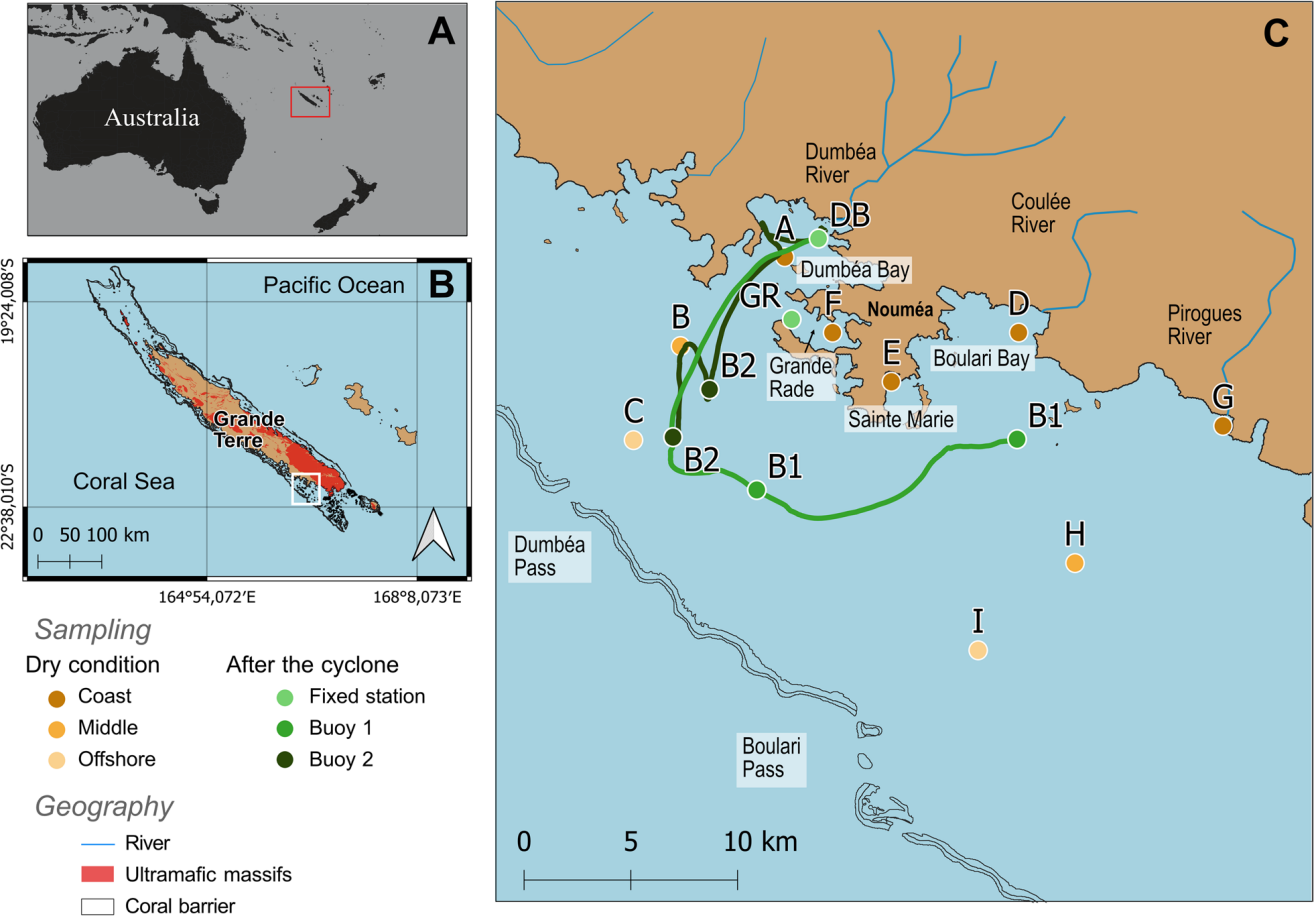
**A** Map of the New Caledonia archipelago in the South Pacific, with **B** a focus of the Grande Terre Island and **C** the bays studied. The stations sampled in the dry seasons (corresponding to September 2019 and December 2020 campaigns with gradient yellow labels) and after the cyclone passage (green labels), with fixed stations and the trajectories of the drifting buoys (B1 and B2), are represented

The New Caledonia archipelago is located, between the 18th and 23rd parallel, at the southern edge of the tropical zone in the Southern Hemisphere in the Pacific Ocean (Fig. 1A). The climate comprises two main seasons, the dry season (generally from June to September) and the wet season (generally from December to March). During austral summer, tropical depressions and cyclones can trigger sudden and intense rainfall [40]. This territory resulted from a non-volcanic geological formation caused by tectonic plate convergence during the Eocene, which resulted in one of the largest ophiolitic complexes on earth [50]. The progressive erosion of the ultramafic rock of the territory induced the transfer of metallic compounds to the lagoon, causing high concentrations and potential bioavailability of metals in the sea [41, 51, 52]. Rivers are vectors of matter toward the lagoon and have a low annual average flow rate but can increase massively after intense rainfall [53].

Our study area was centered in the SW lagoon of the Grande Terre of New Caledonia (Fig. 1B). The quantity of precipitation is about 5 mL d^−1^ annually [54], but this depends on the season, with almost 70% of total rainfall generally occurring in less than 15% of the year, mostly during the wet season [55]. The SW region of the lagoon is under the influence of three main rivers, namely, the Dumbéa, Coulée and Pirogues Rivers, and two connections to the open sea through the Dumbéa and Boulari passes (Fig. 1C). Watersheds are exposed to various land uses that result in different types of anthropogenic pressures on estuaries and adjacent bays (described in Additional file 1).

### Sampling strategy

To investigate the effect of a cyclone on the study site, an *ad-hoc* sampling strategy was designed first to assess the baseline ecological context in a low river input regime during the dry season and then to identify the changes in hydrological and biological variables after a cyclone passage (Additional file 2: Table S1).

To establish baseline ecological conditions, samples were collected spatially during 2 field campaigns corresponding to different periods of the dry season, distinguishable on the basis of sea water temperature. They were carried out from September 23th to 25th, 2019, and from December 8th to 10th, 2020, at 9 stations (Fig. 1C). The cumulative rainfall over 15 days prior to these campaigns was 4.8 mm and 2.4 mm at the Dumbéa meteo station in September and December, respectively (Additional file 2: Fig. S2A, B). Five coastal stations (A, F, E, D, G) corresponded to different types of anthropogenic pressures (Additional file 1), and the remaining 4 stations (B, C and H, I) corresponded to middle- and offshore areas from the Dumbéa (A) and Pirogue (G) river mouths, respectively (Fig. 1C).

**Fig. 2:**
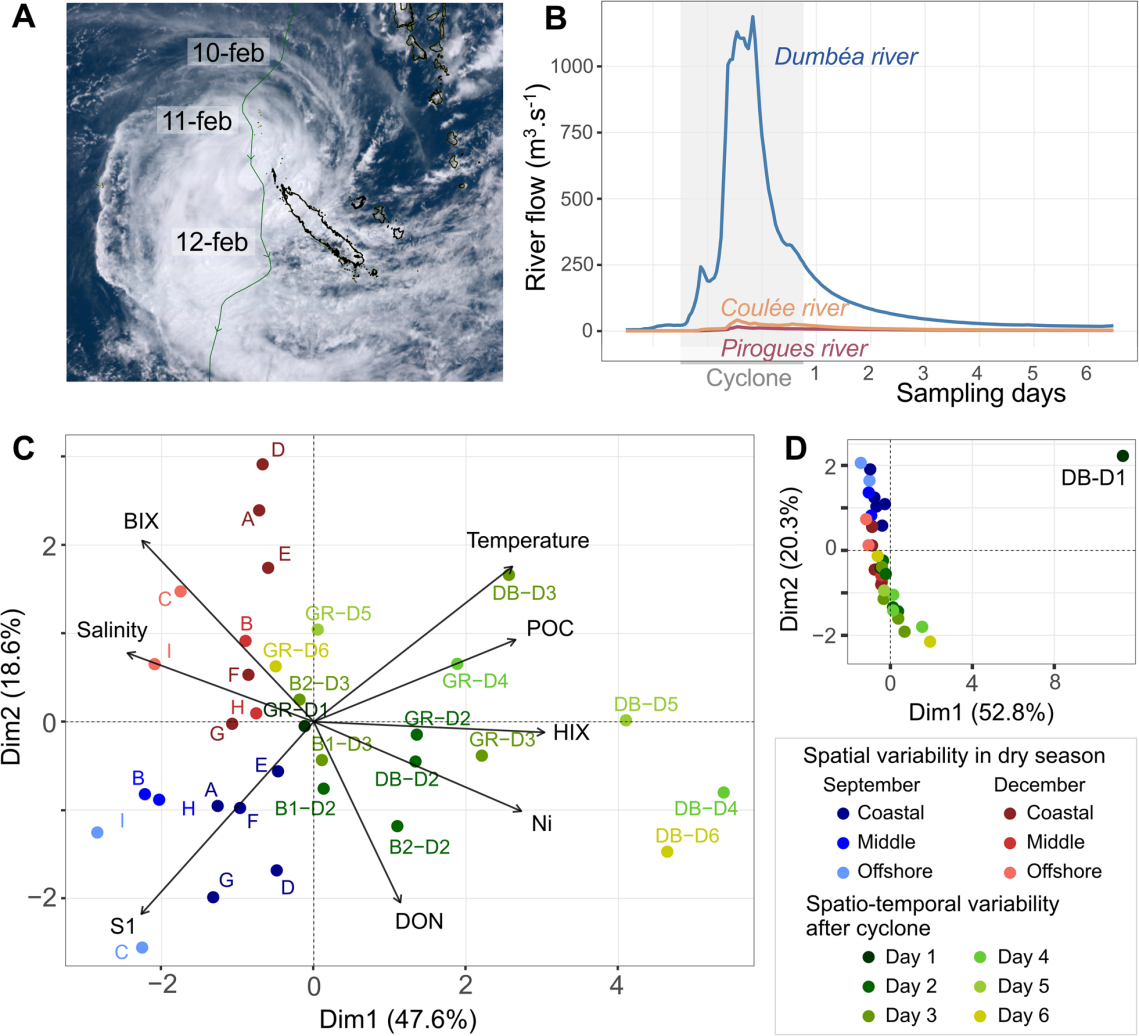
**A** Trajectory of Uesi cyclone (Photography from Hinawara 8 satellite: https://himawari8.nict.go.jp/). **B** Dumbéa River flow during and after the cyclone passage, including the 6 days of sampling, and in relation with the other rivers considered in this study. **C** PCA performed on Euclidian distance and based on eight selected environmental descriptors (Temperature; Salinity; Ni: Particulate nickel concentration; HIX: Humification Index of FDOM; BIX: Biological Index of FDOM; S1: Molecular weight of CDOM; DON: Dissolved Organic Nitrogen; POC: Particulate Organic Carbon) and after **D** exclusion of the outgroup samples collected the first day after the cyclone passage at Dumbéa Bay (DB-B1). The data variance for the two first dimension of the PCA analyses are indicated on the axes. The different sampling stations are labeled according to the sample campaign and in color tones representing either the coastal-off shore gradients (dry season) or the sampling days sampled after the cyclone (D1-D6) in the different fixed point of the studied bays (DB: Dumbea bay; GR: Grand Rade) and the buoys (B1 and B2) trajectories

The cyclone effect was studied during a third campaign from February 12th to 17th, 2020, after the passage of the Uesi cyclone. It occurred from February 10th to 12th and was the first cyclone that impacted New Caledonia during the 2019–2020 cyclonic season. It was considered a magnitude 3 phenomenon (from the Saffir–Simpson scale following Dare and Davidson [56]) at 5:00 am (local hour) on February 11th, 2020, when the wind gust reached 170 km h^−1^. The trajectory of the cyclone followed a north‒south direction (Fig. 2A) and was closer to the territory at 5:00 pm (local hour) on February 11th, 2020, at less than 100 km of the Belep Islands [57]. The south of Grande Terre was more affected by heavy rainfall than by wind. Two sampling strategies were deployed the last day of the cyclone passage, on February 12th. First, two stations (DB and GR) (Fig. 1C) were sampled daily for 6 days to study the microbial temporal dynamics at these sites. Second, the plume of the Dumbéa River was followed by a Lagrangian sampling strategy with 5 drifting buoys (PacificGyre^®^) released once per day from the DB station between February 12th and 16th. Only the two first buoys (B1 and B2) released on February 12th and 13th drifted out of the bay, and the 3 others ran aground because of the low flow, proving that river flow was high enough only mostly during the first two days after the cyclone (Fig. 2B). Samples were collected daily for two days after each buoy release and along the trajectory of both buoys (Fig. 1C).

### Meteorological and hydrological data

Daily precipitation, wind speed and direction were provided by the Meteo France Agency from 3 stations located in cities near our study sites (Nouméa, Tontouta, Dumbéa) (Additional file 2: Fig. S2B). The river flow rates at the water gauging stations of the Dumbéa-East, Dumbéa North, Couvelée, Pirogues and Lembi Rivers were provided by the DAVAR agency (Direction des Affaires Vétérinaires, Alimentaires et Rurales). The few missing data (Coulée River) and the propagation of all river flows were calculated [58] to obtain the flow rates at the river mouth.

### Hydrological and biochemical analyses

At all sites, salinity, temperature, depth and turbidity were measured in situ at 0–2 m of the water column using a SBE19plusV2 CTD (Sea-Bird^®^). An electrode Sentix 41, connected to the WTW3320 case, was used to measure pH. For biogeochemical and biological analyses, 20 L of sea water was collected at each station at 1 m depth.

The ammonium (NH_4_^+^) concentration of unfiltered water was measured by fluorescence using a Trilogy Laboratory Fluorimeter (Turner designs) [59]. Silicate (Si(OH)_4_) concentrations were determined on filter samples of 0.45 µm using a hydrophilic syringe, [60, 61]. Soluble reactive phosphate (SRP), nitrite (NO_2_^−^), nitrate (NO_3_^−^) (summed as NO_x_) and dissolved organic nitrogen (DON) were analyzed from filters (Whatman^®^ grade GF/F) precombusted at 450 °C for 1 h. SRPs were analyzed following the Hansen and Koroleff method [62]. NO_x_ were measured by colorimetry on an Autoanalyser SEAL [63, 64] after reduction of NO_3_^−^ to NO_2_^−^ [65]. Finally, DON was analyzed by continuous flow after wet oxidation and mineralization [66].

The concentrations of chlorophyll *a* (Chl *a*) and phaeophytin were determined before and after acidification with 90% acetone extracts from water filtered on Whatman^®^ grade 0.7 µm GF/F by fluorescence [59].

For dissolved organic matter (DOM), the optical properties of colored and fluorescent DOM were used as quality and origin proxy. For CDOM, absorbance at 350 nm (A_350_) is considered as a proxy of terrestrial input in water [67] and the spectral slope derived from a_275_ and a_295_ (S1) indicated the origin, molecular weight and photochemical sensitivity [68, 69]. The index of humification (HIX) and the autotrophic productivity (BIX) were calculated with the fluorescence properties of CDOM [70, 71].

Particulate concentrations of nickel (Ni), cobalt (Co), manganese (Mn), iron (Fe), chromium (Cr), copper (Cu) and zinc (Zn) were measured by inductively coupled plasma spectrometry (ICP‒OES Varian 730-ES) after mineralization. The particulate organic carbon (POC) was measured by spectroscopy after preheat and filtering of water. More details of the method are available in Additional file 1.

### Genetic analyses

Sea water samples (between 4 and 15 L) were sequentially filtered using 20 µm and 3 µm (Whatman^®^ GF/F) filters using a peristaltic pump (Alexis^®^). Then, about 1 L of the prefiltered water sample was passed (in two replicates only for the September 2019 cruise) through a 0.2 µm filter, avoiding filter saturation. We ended up with 114 samples for protists (34, 35 and 45 for the size fractions > 20, 20–3 and 3-0.2 µm, respectively) and 114 for bacteria (34, 35, 45). For simplicity, in this study, we considered that according to the filters used, we separated micro-protists (> 20 µm), nano-protists (20–3 µm), pico-protists (3–0.2 µm), particle-associated bacteria (PAB, > 20 µm and 20–3 µm) and free living bacteria (FLB, 3–0.2 µm).

The composition of microbial communities was assessed using a metabarcoding approach [72–74] of eDNA extracted from filters. eDNA was extracted using the DNA extraction kit Nucleospin Plant II Mini (Macherey–Nagel, Hoerdt, France) following the manufacturers’ protocol. The extracted eDNA concentration was verified with a Qubit 4 (Thermo Fisher Scientific). The amplified regions were chosen for their consistent presence and variability in the target organisms, allowing a sufficient precise taxonomic assignment for a community-level study [75, 76]. Moreover, the V4-18S used for protists showed a good characterization of community diversity and better representation in reference databases than others marker region [77, 78], and the region V4-V5 of 16S rDNA, for bacteria, was recommended by the earth microbiome project (https://earthmicrobiome.org/protocols-and-standards/16s/). Amplification was performed by PCR in triplicate for each DNA extract. The V4 18S marker was targeted using the following set of primers and the sequencing adapter (from GeT-Biopuces platform): TAReuk454FWD1 (5′ CTT TCC CTA CAC GAC GCT CTT CCG ATC TCC AGC ASC YGC GGT AAT TCC 3′) and TAReukREV3 (5′ GGA GTT CAG ACG TGT GCT CTT CCG ATC TAC TTT CGT TCT TGA TYR A 3′) [75]. The V4–V5 16S marker was targeted using the primers 515F-Y (5′ CTT TCC CTA CAC GAC GCT CTT CCG ATC TGT GYC AGC MGC CGC GGT AA 3′) and 926R (5′ GGA GTT CAG ACG TGT GCT CTT CCG ATC TCC GYC AAT TYM TTT RAG TTT 3′) [76]. The PCR mix recipe and program was adapted from [76, 79], and are detailed in Additional file 1. PCR blanks were used to identify potential contaminations in the samples. Amplification results were checked by gel electrophoresis (Additional file 1: Figs. S1–S7). Sample triplicates were pooled and sent to the GeT-Biopuces platform (INSA, Toulouse, France) for Illumina MiSeq sequencing with a standard kit V3 (2 × 250 bp). Raw data are available on Sextant Ifremer, at 10.12770/26840434-8354-4856-9d5e-e562c8de7252.

Raw data were treated using SAMBA (Standardized and Automated MetaBarcoding Analyses) open-source pipeline developed by the SeBiMer (Ifremer’s Bioinformatics Core Facility) (https://github.com/ifremer-bioinformatics/samba). In summary, the workflow allowed us to clean, filter, trim and merge reads using QIIME2 [80] and DADA2 [81], to cluster ASV sequences with dbOTU3 [82] and to remove contamination with microDecon [83]. The ASV assignments were performed with the PR^2^ database (version 4.13.0) for protists [84] and SILVA (version 138) for bacteria [85]. Rarefaction curves were checked on both markers (Additional file 2: Fig. S1) and CSS (Cumulative Sum Scaling) data normalization was performed to avoid sequencing depth bias [86]. In the 18S dataset, ASVs assigned to multicellular organisms (Metazoa, Streptophyta, Florideophyceae and Ulvophyceae) were removed to consider only unicellular micro-eukaryote (protist) diversity. In the 16S dataset, the archaea, mitochondrial and chloroplast sequences were removed to keep only bacterial organisms. In the resulting table, ASVs with fewer than 2 reads were removed to limit sequencing artifacts [87]. Finally, after database cleaning, sequencing data represented 2,404,149 reads in total, with 10,994 ± 4041 reads/sample and 10,094 ± 3274 reads/sample in the 18S and 16S tables, respectively. All reads were assigned to 2861 protist ASVs and 1834 bacterial ASVs in the overall samples of our study.

Protist and bacterial functional classification focused on trophic type and was based on the taxonomic assignments obtained following the functional protist database of Ramond et al. [79] and the work Ramond et al. [88] (detailed in Additional file 1).

### Hydrodynamic model

The MARS3D numerical model used here has been described in detail by Lazure and Dumas [89]. In short, it is a standard model based on classical assumptions (Boussinesq approximation, hydrostatic balance assumed) that leads to a set of equations that is solved using finite difference techniques in a sigma coordinate framework. The resolution is based on mode splitting, as in Blumberg and Mellor [90]. The turbulent eddy diffusivity and viscosity are assessed using the k-ε turbulence closure. To model the SW lagoon of New Caledonia, two different levels of nesting were required and were coupled using the AGRIF procedure [91]. The first level described the open boundary conditions of the lagoon, including regional currents and astronomical and meteorological tides at a resolution of 1500 m. It is forced by a sea level signal harmonically composed of FES2012 [92] and by the MERCATOR model [93], providing realistic conditions of sea level anomalies, regional currents and water properties (temperature and salinity). The bathymetry of the grids was obtained from the French Oceanographic and Hydrographic Service (SHOM). The detailed inner hydrodynamic model has a spatial resolution of 300 m. It encompassed the entire SW lagoon, rim and external slope. On the vertical axis, 50 sigma layers were distributed to represent both the bottom and the surface boundary layers. The atmospheric forcings were computed thanks to the bulk formulae following Luyten and De Mulder [94], and the wind drag coefficient was computed following Charnock's relation [95]. These formulae require wind velocity at 10 m, pressure at sea level, relative humidity and air temperature at 2 m, and finally fractional cloud cover. These meteorological conditions were obtained from ERA5 reanalysis [96]. Eventually, river runoff was prescribed at the outlet of the three main rivers of the area (Fig. 1C).

### Statistical analyses

All statistical analyses were performed with R Statistical Software v4.2.1 [97]. A total of 21 variables were chosen to characterize the environment. A Spearman’s correlation matrix was performed to avoid autocorrelated parameters and select key variables in the following ecological computations. Principal component analysis (PCA) was performed on the 8 normalized key environmental parameters using the Euclidean distance. Alpha diversity was estimated with the number of ASVs using different sample classifications (campaigns, spatial gradient, size class, and days after the cyclone). The influence of several qualitative factors (campaigns, size class) on the composition of communities was tested with a PERMANOVA. Beta diversity was studied with multivariate analyses using the Bray‒Curtis distance on the read/ASV matrix. Non-metric multidimensional scaling (nMDS) was performed to understand the structuration of the community by sample clustering. Distance-based redundancy analyses (db-RDA) allowed us to constrain the community structure with the same environmental parameters used in the PCA. It helped to understand the relation of the size-classed communities of both protists and bacteria with the environment. ASV relative abundances were grouped to the genus level in analyses considering genus temporal dynamics. To study the temporal dynamics of communities after cyclonic perturbation, ecological trajectories of different size-classed communities were performed using principal coordinate analysis (PCoA) [98] on ASV microbial community datasets.

## Results

Based on the combination of hydrological, meteorological, chemical and biological data, this study showed the impact of river runoff on coastal subtropical ecosystems after a tropical storm. This effect was evaluated in comparison with ecological conditions occurring in the dry season.

### Hydrological and biogeochemical ecological descriptors

To assess the variability of the ecosystem, 8 ecological descriptors, over the 21 measured, were chosen to represent environmental variabilities: Salinity, water temperature, POC (Particulate Organic Matter), Ni (particulate Nickel), S1 (proxy of molecular weight of CDOM), HIX (Humification Index), BIX (Biological Index) and DON (Dissolved Organic Nitrogen) (detailed in Additional file 2). The whole dataset is available in Additional file 3. The passage of the Uesi cyclone over the territory of New Caledonia (Fig. 2A) caused a strong increase in the flow of the Dumbéa River (Fig. 2B). The hydrological and biogeochemical conditions of DB varied considerably (Fig. 2C, D), as shown by the separation of sample DB-D1 in the PCA (Fig. 2D). After the removal of this station, the 8 key parameters explained 65.2% of the environmental variability of the remaining samples and allowed a separation between the dry season (September and December) and the post-cyclone water conditions (Fig. 2C).

### Ecological variability during the dry season

#### General ecological patterns

**Fig. 3:**
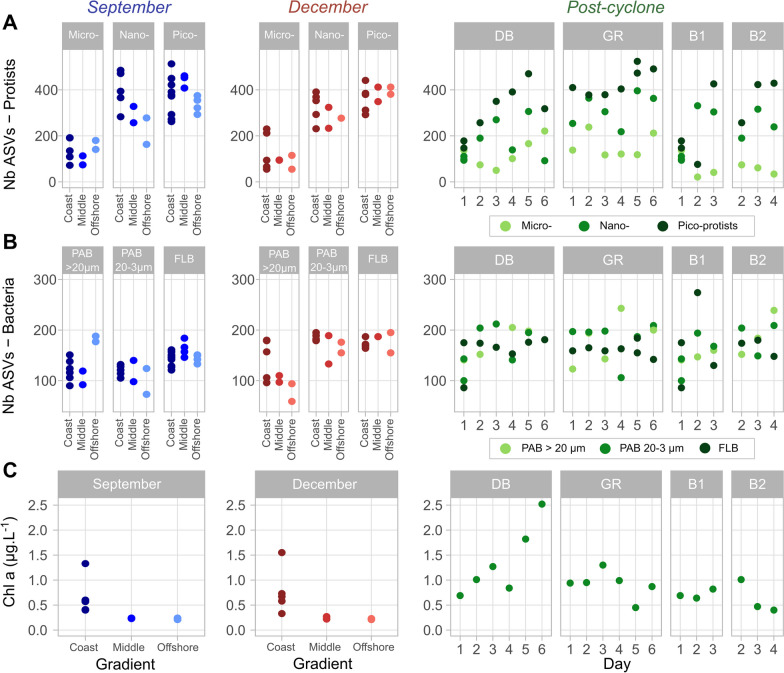
ASV (Amplicon Sequence Variant) richness of **A** micro-, nano- and pico- protists, **B** bacterial communities and **C** chlorophyll a concentration as proxy of phytoplankton abundances across the board-coast gradient of the dry season (September and December), and along the 6 days following the Uesi cyclone in two bays [Dumbea bay (DB) and Grande Rade (GR)] and in the river plume (Buoys 1 and 2)

**Fig. 4:**
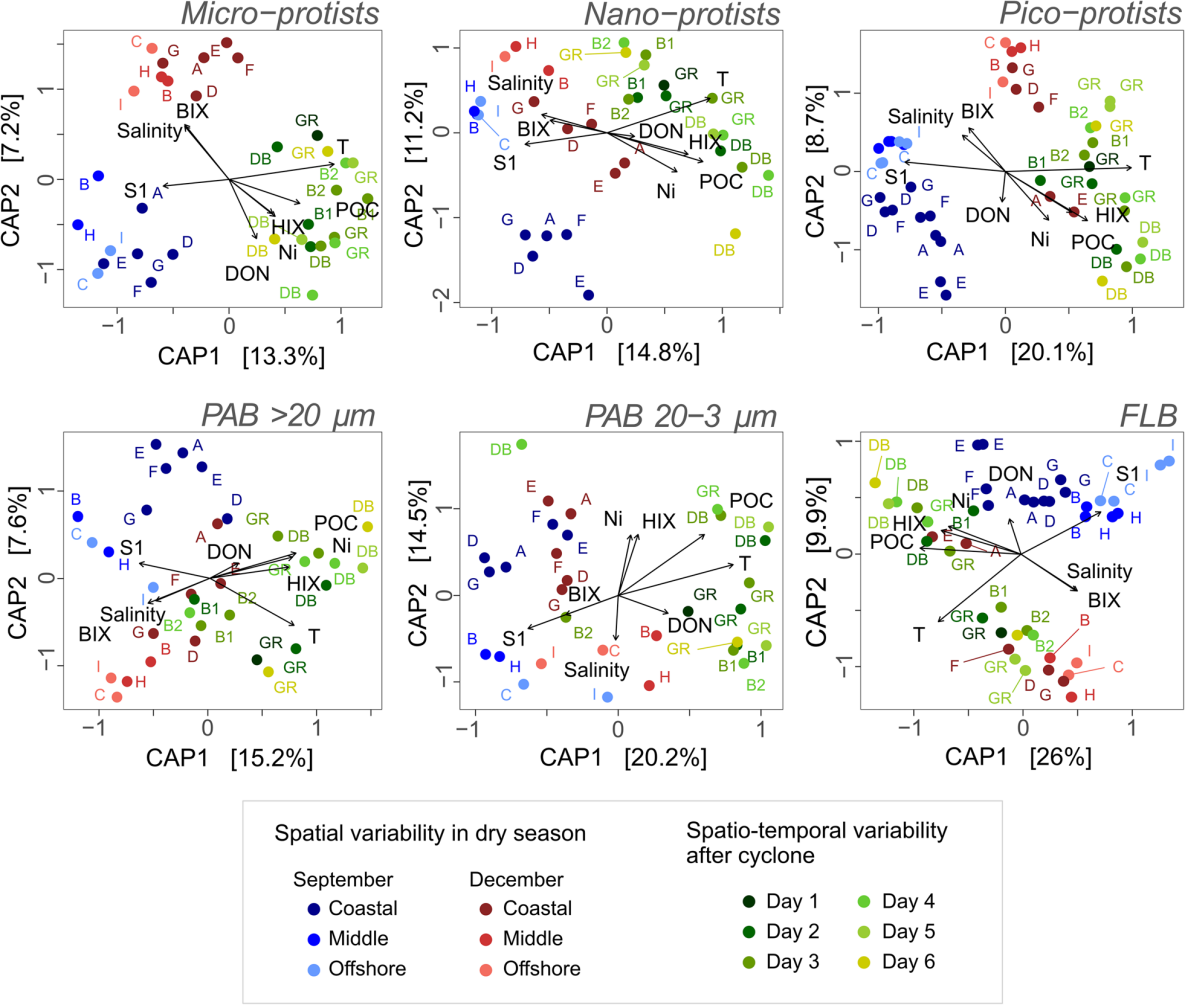
db-RDA of protist and bacterial communities of micro-, nano- and pico-protists in relation with 8 selected explanatory hydrological and biogeochemical variables (T: Temperature; Salinity; Ni: Particulate nickel concentration; HIX: Humification Index ofFDOM; BIX: Biological Index of FDOM; S1: Molecular weight of CDOM; DON: Dissolved Organic Nitrogen; POC: Particulate Organic Carbon). The ordination is based on the Bray–Curtis distance

September and December campaigns occurred during dry conditions, with little rainfall recorded 15 days before sampling (Additional file 2: Fig. S2A). Some similar patterns can be identified between the two dry season campaigns. For instance, seawater salinity stayed stable with a range between 35.4 and 35.9 during both campaigns. However, the microbial communities were strongly diversified by their size classes for both protists (Additional file 2: Fig. S4A) and bacteria (Additional file 2: Fig. S4B), justifying the separation of communities according to size classes in the following analyses. Indeed, microbial richness (Fig. 3) and community compositions (Fig. 4) varied according to the size classes. For both dry season campaigns, pico-protists were three times richer in diversity than micro-protists (382 ± 72 ASVs in pico- vs. 134 ± 46 ASVs in micro-protists in September, 374 ± 48 in pico- vs. 113 ± 65 in micro-protists in December) (Fig. 3A). A general microbial community structure can be identified during the dry season at a high taxonomic level (Additional file 2: Figs S5, S6). At the class level, micro-protists were largely dominated by Dinoflagellata (73 ± 17%) and to a lesser extent by Ochrophyta (13 ± 9.6%). Pico- and nano-protists were more diverse and dominated by Dinoflagellata (50 ± 13% and 59 ± 21%, respectively, in September and December), Ochrophyta (6.3 ± 4.6% and 16 ± 17%), Chlorophyta (15 ± 12% and 4.8 ± 6.0%), Haptophyta (4.6 ± 2.0% and 4.6 ± 2.9%) and Cryptophyta (3.1 ± 2.9% and 2.2 ± 2.9%). Bacterial communities also showed different phylum compositions depending on size classes, which are a proxy of bacterial association with organic and inorganic particles (PAB, 20–3 µm and > 20 µm size classes) or not (FLB, 0.2–3 µm size class). The dominant PAB belonged to Bacteroidota (31 ± 14% and 27 ± 11%, respectively, in September and December), Proteobacteria (24.4 ± 10% and 16 ± 12%), Cyanobacteria (21 ± 12% and 20 ± 9.5%), Planctomycetota (15 ± 11% and 15 ± 9%), Verrucomicrobiota (7.7 ± 7.4% and 3.6 ± 2.6%) and Actinobacteriota (1.3 ± 1.0% and 2.0 ± 1.2%). Instead, FLB showed less phylum diversity with a dominance of Proteobacteria (50 ± 7.2%), Actinobacteriota (4.9 ± 1.2%), Bacteroidota (15 ± 3.3%) and Cyanobacteria (25 ± 7.8%). In terms of trophic functional biodiversity, all protist communities were similar during the dry season, with an average of 55 ± 16% photoautotrophic/mixotrophic organisms. Nano- and micro-protists slightly exceeded this average value, with 59 ± 9.6% and 58 ± 14%, respectively, whereas pico-protists showed lower average values (42 ± 13%), indicating that heterotrophic organisms were more abundant in the smallest protist size fraction. Bacterial communities showed a clear dominance of heterotrophic taxa, with an average of 74 ± 7.9% for FLB, 71 ± 6.9% for PAB of 20–3 µm and 85 ± 7.6% for PAB of > 20 µm.

#### Variability within the dry season

Despite these similarities, the two campaigns showed some specificities in terms of environmental conditions (Fig. 2C) and microbial community composition (Fig. 4, Additional file 2: Figs. S5, S6) mostly explained by BIX, S1, DON and temperature. December conditions showed higher mean values across stations in comparison to September for BIX (+ 0.25 ± 0.28) and temperature (+ 3.8 ± 0.70 °C) and lower values in S1 (− 0.008 ± 0.007 nm^−1^) and DON (− 1.4 ± 1.6 µmol L^−1^). The alpha diversity of bacterial communities followed this pattern, with a richer diversity in December (on average + 29 ± 17 ASVs of FLB and + 60 ± 17 ASVs of PAB of 20–3 µm) (Fig. 3B). This trend was not visible for protist communities, with the number of ASVs not significantly different between the two campaigns (*p* value > 0.05). Both protist and bacterial communities showed some significant differences when analyzed in terms of ASV composition (PERMANOVA < 0.05), which were partly explained by environmental parameters (Fig. 4). For instance, Dinoflagellata was 16 ± 16% higher in December among the micro-protists. A decrease in photoautotrophic/mixotrophic taxa in some coastal stations (D, E, F and G) of pico-protists was also recorded in December (− 16 ± 6.1%), notably related to a reduction in Ochrophyta (− 8.0 ± 7.2%) and Chlorophyta (− 13 ± 8.1%) organisms.

#### Spatial gradient

Spatial variability was evident during the dry season along the coastal-offshore sampled gradients. The S1, HIX, Ni and POC values explained most of this spatial variability (Fig. 2C). Both sampled transects showed a reduction in HIX (− 0.18 in September and − 0.27 in December), Ni (− 1.86 and − 1.36 µg L^−1^) and POC (− 0.07 and − 0.1 mg L^−1^) from the coast to offshore stations. During both campaigns, coastal biodiversity was higher for some size fractions (Fig. 3). Over the whole sampling area, 620 protist ASVs and 427 bacterial ASVs were found only in coastal stations, while 325 protist ASVs and 265 bacterial ASVs were found only in middle or offshore stations. The coastal-offshore biodiversity gradient corresponded to community taxonomic composition variations and trophic functional biodiversity changes (Additional file 2: Figs S5, S6). For instance, this was illustrated by the higher proportion of Dinoflagellata, Ochrophyta and Cyanobacteria in coastal stations (more detailed in Additional file 2). The higher proportion of photoautotrophs was consistent with the higher Chl *a* concentration in coastal stations (0.72 ± 0.40 µg L^−1^), while the middle and offshore stations had concentrations approximately three times lower (0.23 ± 0.02 µg L^−1^) (Fig. 3C).

#### Coastal heterogeneity

In addition to the spatial gradient, the biotic and abiotic components of coastal stations showed more heterogeneity than middle and offshore stations (Figs. 2C, 4). For example, the concentration of Ni ranged between 0.46 (station E) and 3.35 (D) µg L^−1^ in September and between 0.11 (D) and 2.26 (F) µg L^−1^ in December. In terms of community composition, coastal heterogeneity was particularly evident for the nano-protists, where Dinoflagellata ranged from 21 to 49% in September and from 41 to 79% in December and Ochrophyta varied from 12 to 57% in September and 4.8–52% in December (Additional file 2: Figs. S5, S6). Fungi represented more than 1% of the relative abundance in September at stations D, E and F but were scarcer in December. More specifically, some taxonomic groups were found only in certain coastal stations. The G station was the only coastal station where Holophagae (Acidobacteriota) were found in the PAB > 20 µm fraction, despite at low proportion (0.07% in December and 0.03% in September).

### Impact of river runoff after the cyclonic event

#### Physical disturbance

**Fig. 5:**
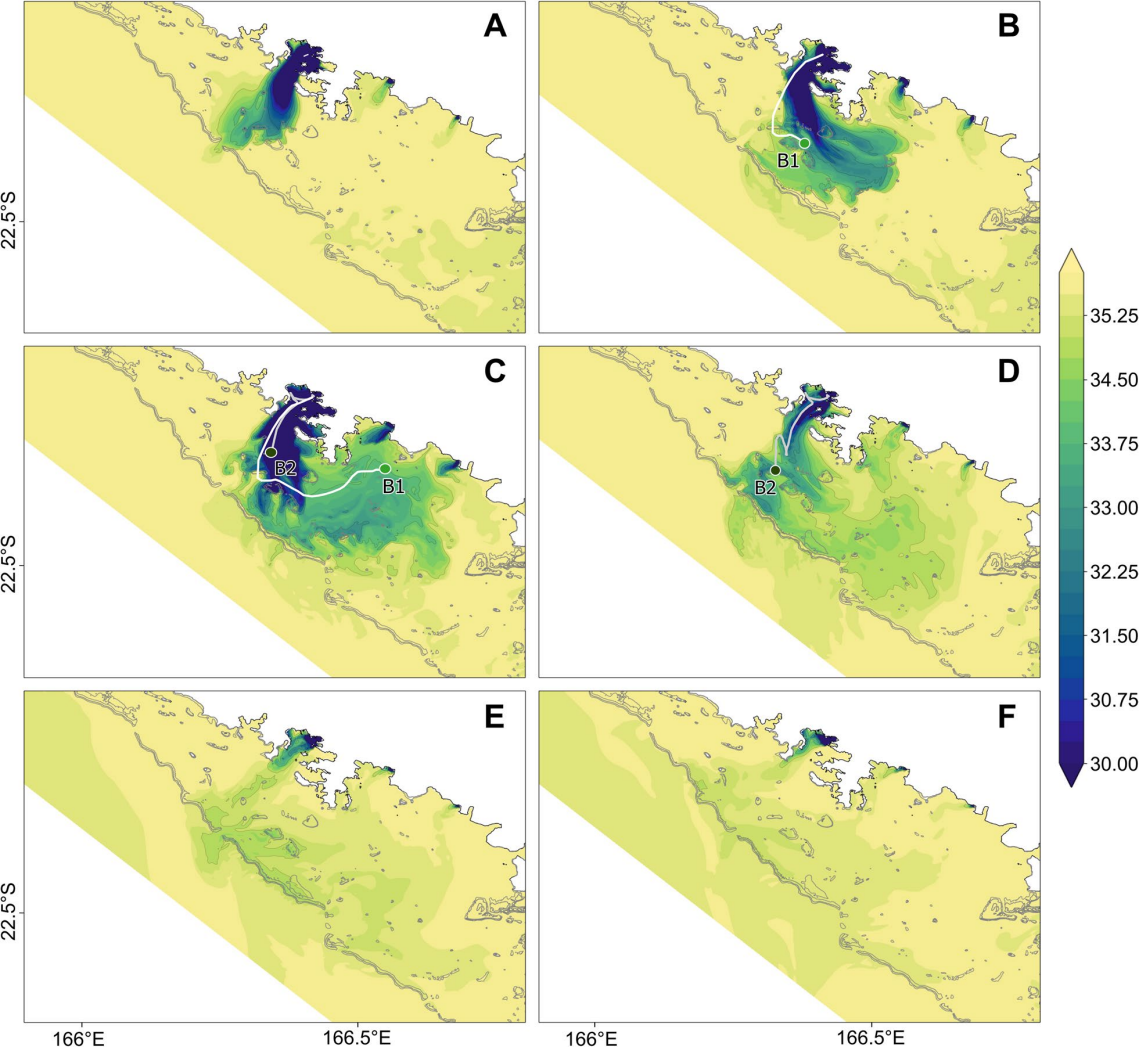
Modelled sea surface salinity at each sampling time after the cyclone (**A**: Day 1, **B**: Day 2, **C**: Day 3, **D**: Day 4, **E**: Day 5, **F**: Day 6)

Between the February 10th and 12th 2020, Uesi generated massive rainfall events that represented approximately 14% of the annual cumulative precipitation recorded in 2020 (January to mid-December) at the Dumbéa station (Additional file 2: Fig. S2A, B). During this period, the lagoon received a large amount of freshwater from rivers. The Dumbéa River's flow increased 40-fold in less than 24 h and reached a maximum river flow value of 1188 m^3^ s^−1^ on February 11th (Fig. 2B). Comparatively, the Coulée and Pirogues watersheds were less impacted by heavy rainfall, and their river flows reached maximums of 41.9 and 15.9 m^3^ s^−1^, respectively, during the cyclonic event. Realistic simulation of this event provided a view of the spatiotemporal extension of the plumes. Globally, due to the backing winds that occurred during and after the event, we can consider three main phases. During the first phase (Fig. 5A), the plumes spread cross-shore in the direction of the barrier reef due to the light north-easterly winds and the huge flow just after the passage of Uesi. Then, the major plume (issued from the Dumbéa River) followed a southeast direction (Fig. 5B, C) and was mainly influenced by the northwestern wind conditions (Additional file 2: Fig. S2C). On Day 2, the plume impacted the GR station (Fig. 5B) and showed a maximum spatial extent on day 3 when it propagated across the whole study site (Fig. 5C). Finally, from days 4 (Fig. 5D) and 5 (Fig. 5E), because of the return of trade winds and the decrease in flows, the plume started to dilute within the lagoon, and on day 6, the influence of this flash flood was nearly no more visible on the surface salinity signal except at the back of DB (Fig. 5F). The modeled plume issued from the Dumbéa River was in very good agreement with the trajectories obtained from our drifting buoys B1 and B2 (Fig. 1C).

#### Daily perturbation

**Fig. 6:**
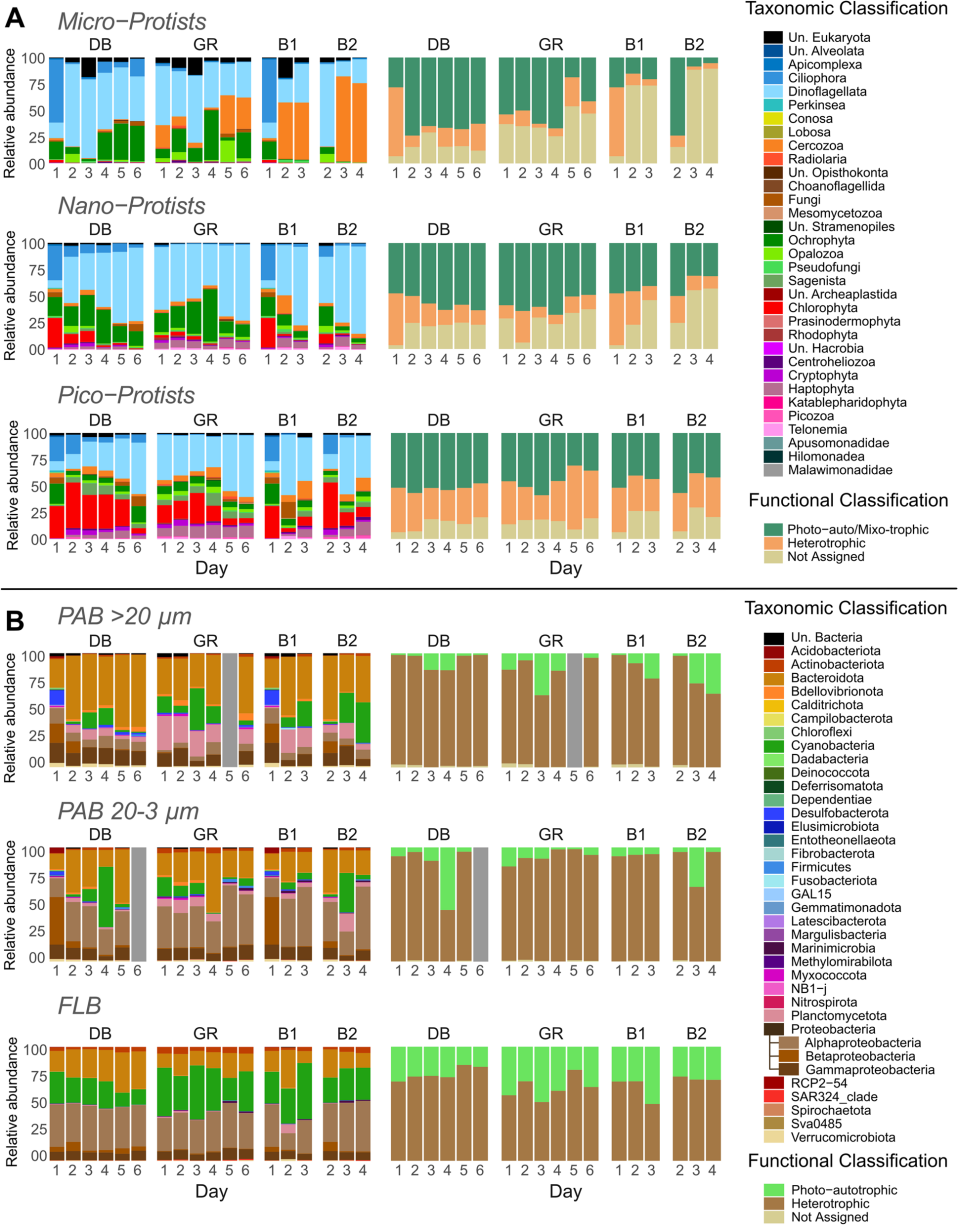
Taxonomical and functional community composition of **A** micro-, nano-, and pico-protists and **B** PAB and FLB during the rainy season campaign of February 2020. Community composition are shown during the 6 days following the Uesi cyclone in two bays [Dumbea bay (DB) and Grande Rade (GR)] and for samples collected along the trajectories of buoys B1 and B2. Protist classes and bacterial phyla are represented in relative abundance (%). For Proteobacteria the classes of Alpha-, Beta- and Gamma-Proteobacteria are also represented. (Unassigned: Un.)

**Fig. 7:**
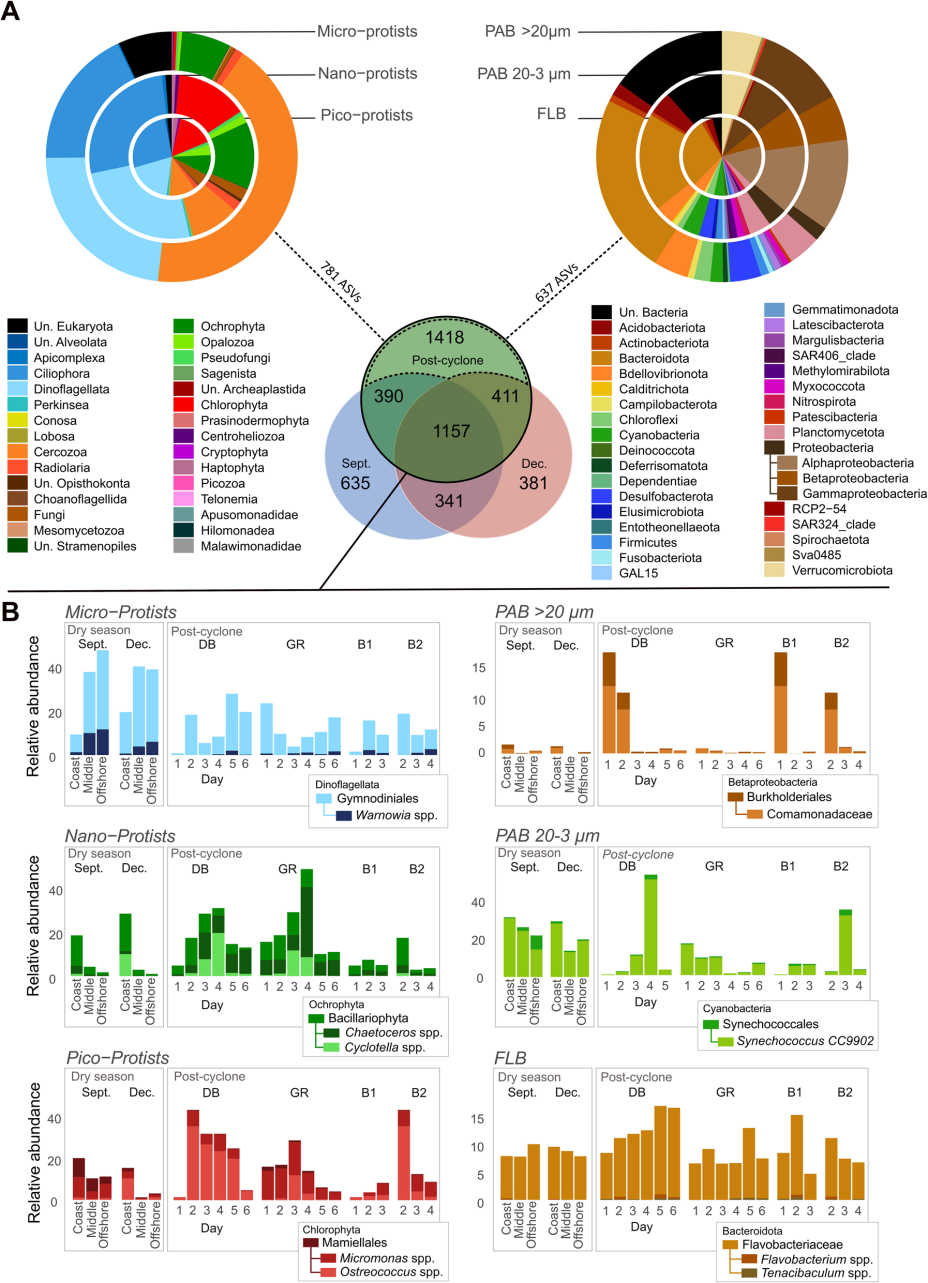
**A** ASVs found only after the cyclone during the February 2020 campaign, in protist and bacterial communities. **B** Spatio-temporal evolution of relative abundance of significant taxonomic groups in the size classes sampled, in two bays (DB and GR) and in samples collected along the buoys trajectories. These values are compared with relative abundances found during samples collected in the dry season (September and December). (Unassigned: Un.)

This massive runoff disturbance caused daily variations in chemical parameters and microbial community composition in the DB and GR bays, both of which were affected by river runoff (Figs. 2C, 4, 6). All environmental parameters measured showed strong changes, either sharply increasing (e.g., + 223 µmol L^−1^ of Si(OH)_4_ on DB on Day 1) or decreasing (e.g., − 33 salinity units) (Fig. 2C and Additional file 2: Fig. S7). A total of 1418 ASVs were found only after the Uesi cyclone (February campaign, all protists and bacteria sampled pooled) (Fig. 7A), corresponding to 43% of the ASV biodiversity of all the datasets. Among those new ASVs, most belonged to Dinoflagellata (122 ASVs in all 3 protist communities), Ochrophyta (68 ASVs), Proteobacteria (186 ASVs in all bacterial communities) and Bacteroidota (136 ASVs). Most of the ASVs present after the cyclone passage were not primarily dominant; for example, the 122 new Dinoflagellata ASVs showed an average relative abundance of 3.8 ± 4.8%. A significant portion (88 protist and 84 bacterial ASVs) were not assigned to any known organisms according to the PR^2^ and SILVA databases. When annotated with the BLAST database (v2.13.0), only 5 ASVs sequences were associated with unknown soil organisms.

The first sampled day after the cyclone passage (Day 1) was marked by a net change in environmental parameters and microbial communities in DB. Salinity dropped to 2.15, and HIX and Ni concentration peaked at 15.1 and 214 µg L^−1^ respectively. Some microbial communities showed a high drop in ASV richness. The highest reduction occurred in the pico-protist community, with a loss of 50% of ASVs compared to the dry season (Fig. 3A). DB was also marked by a net variation in both protist and bacterial community composition in comparison to those found at coastal stations (A, D, E, F and G) during the dry season (Fig. 6A, B). Some proxies of this change were the variations in relative abundance of Ciliophora (+ 21% for pico-, + 31% for nano- and + 38% for micro-protists), Fungi (+ 4.8% for pico- and + 4.1% for nano-protists) (Fig. 6A), Betaproteobacteria (+ 41% for PAB of 20–3 µm and for + 17% for PAB > 20 µm), and Desulfobacterota (+ 2.5% for PAB of 20–3 µm and + 12% for PAB > 20 µm) (Fig. 6B). This increase corresponded to a decrease in the relative abundance of other groups, such as Dinoflagellata (− 34% for pico-, − 42% for nano- and − 52% for micro-protists) (Fig. 6A), Planctomycetota (− 4.5% for PAB of 20–3 µm and -13% for PAB > 20 µm) and Cyanobacteria (− 33% for PAB of 20–3 µm- and − 12% for PAB > 20 µm) (Fig. 6B). Certain groups also appeared on Day 1, such as Perkinsea (2.0% in pico-protists), Malawimonadidae (0.4% in pico-protists) (Fig. 6A), Methylomirabilota (0.8% in PAB 20–3 µm), Elusimicrobiota (0.4% in PAB 20–3 µm), Entotheonellaeota (0.4% in PAB 20–3 µm), GAL15 (< 0.1% in PAB 20–3 µm), and the ASV annotated as RCP2-54 (< 0.1% in PAB 20–3 µm) (Fig. 6B). This taxonomic change corresponded to a variation in trophic diversity in the system, with an increase in the heterotrophic compartment. The heterotrophic taxa proportion increased in protist communities (+ 14 ± 6.6% in pico-, + 29 ± 2.6 in nano- and + 45 ± 6.6% in micro-protists), mainly due to the augmentation of Ciliophora. In PAB communities (+ 27 ± 6.9% in PAB 20–3 µm and + 9.7 ± 5.2% in PAB > 20 µm), it was associated with the reduction in Cyanobacteria relative abundances. Most of these changes in biodiversity could not be ascribed to well-annotated taxa/ASVs, as most of them were only assignable to the class. Within Ciliophora, a single ASV assigned to the Oxytrichidae family dominated in micro-protist community with 41% relative abundance. The FLB community did not show a clear change on the first day after the cyclone.

#### Short-term coastal dynamics

**Fig. 8:**
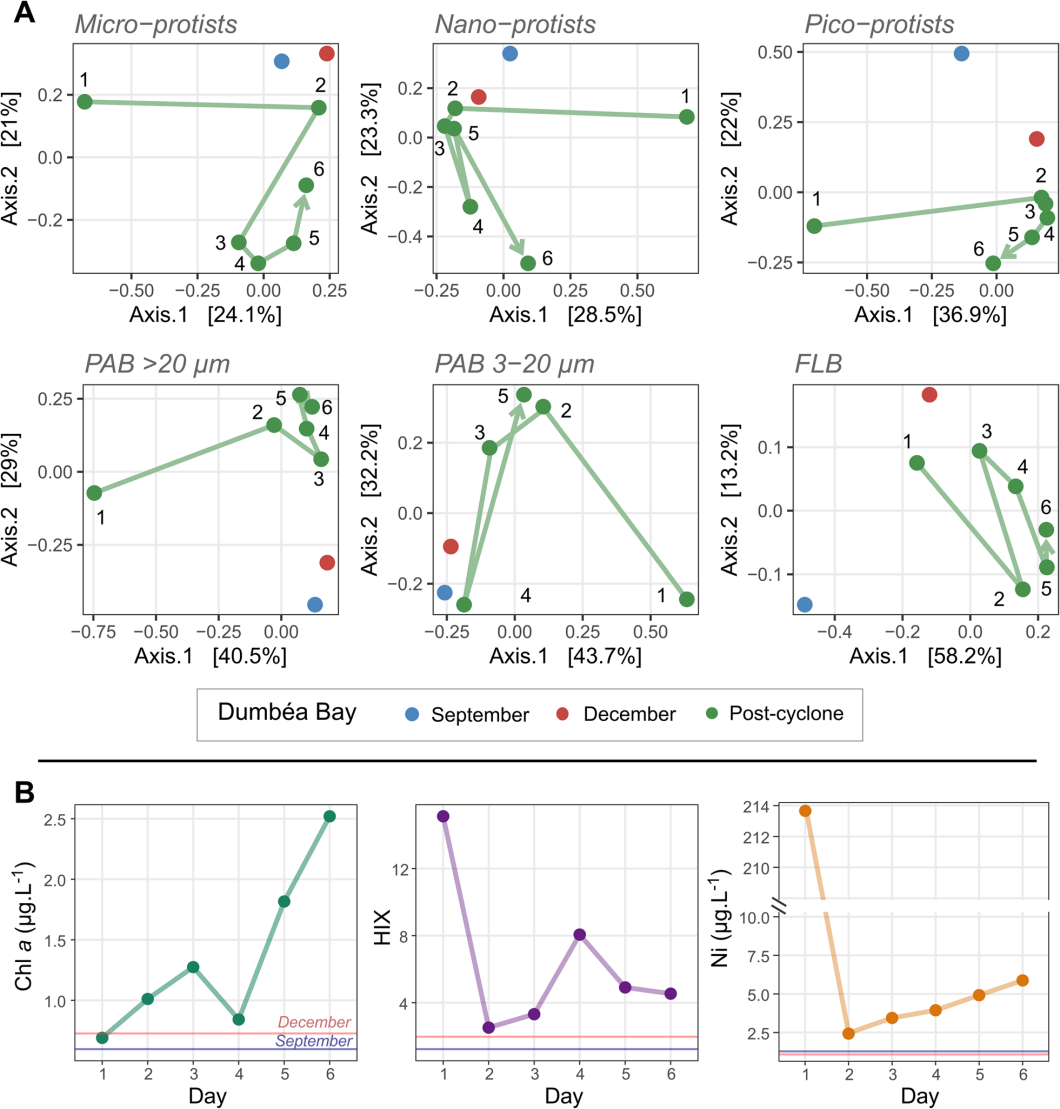
**A** Microbial community trajectories during the 6 days after the cyclone at Dumbéa Bay (DB) for protists and bacteria. Data of station A, sampled during the dry seasons (September and December), are used as reference conditions. **B** Dynamics of selected environmental parameters compared to reference condition of the dry season. Lines are used for this representation supposing that values at DB did not vary significantly during the dry season

After the drastic change on Day 1, the trajectories of the microbial community composition evolved over the following 6 days. The microbiome of the DB showed specific dynamic and important variations in composition in comparison with the dry period (Fig. 8A). Ecological trajectories of microbial communities based at the ASV taxonomic level showed differences between dry season samples of station A in September and December and DB-Day 6 sampled after the cyclone (Fig. 8A). A sharp change in environmental parameters and microbial community composition was observed between days 1 and 2. On day 2, an increase in temperature (+ 3.1 °C), salinity (+ 34), pico- and nano-protists richness (+ 95 and + 89 ASVs), PAB of 20–3 µm richness (+ 75 ASVs) and the proportion of photoautotrophic taxa in micro-protists (+ 46%) was observed. Conversely, a decrease in POC (− 1.5 mg L^−1^), Ni (− 211 µmol L^−1^), HIX (− 13), DON (− 15 µmol L^−1^) and micro-protists richness (− 69 ASVs) was registered. This change between Day 1 and 2 was well represented by some specific taxonomic group, with a high augmentation of Mamiellales in pico-protists (+ 41%) (Fig. 7B).

After Day 2, the environmental parameters and community microbial composition showed specific dynamics. HIX (+ 2), Ni (+ 2.5 µmol L^−1^), Chl *a* (+ 1.5 µg L^−1^), pico- and micro-protist richness (+ 61 and + 147 ASVs) showed an increase until Day 6. In terms of taxonomic composition, Dinoflagellata progressively increased in relative abundance (+ 39% for pico-, + 61% for nano- and + 27% for micro-protist communities between days 1 and 6), whereas Ciliophora (-15% for pico-, − 29% for nano- and − 42% for micro-protist communities) and Chlorophyta (− 25% for pico-, − 26% for nano- and − 2.0% for micro-protists) progressively decreased. Some groups/genera showed specific temporal dynamics during the sampling period (Fig. 7B). For instance, Bacillariophyta in the nano-protist community showed a peak of relative abundance (reaching 31% of relative abundance) after 4 days, with values higher than those found at station A during the dry season (9.0% in September and 8.5% in December). Within Bacillariophyta, on Day 4, two genera were dominant: *Cyclotella* spp. (19%) and *Chaetoceros* spp. (7.9%). Marine *Synechococcus* (Synechococcales) (with dominance of ASVs annotated as *Synechococcus* CC9902) showed the same dynamic in PAB of 20–3 µm, with a peak at 53%, also visible with the augmentation of photoautotrophic bacteria (Fig. 6B). Day 4 was also associated with a peak of NH_4_^+^ (1.22 µmol L^−1^), Si(OH)_4_ (119 µmol L^−1^) and HIX (8) (Additional file 2: Fig. S7). At the end of our sampling period, on Day 6, Dinoflagellata (> 42%) and Ochrophyta (> 14%) dominated the protist communities (Fig. 6A). However, some biological and biogeochemical consequences of the passage of the cyclone were still visible. Higher values of Chl *a* (+ 1.8 ± 0.1 µg L^−1^ compared to station A in the dry season), HIX (+ 2.9 ± 0.4) and Ni (+ 4.7 ± 0.1 µmol L^−1^) were still observed (Fig. 8B). In addition, in comparison with station A in the dry season, a higher proportion of Desulfobacterota (+ 1.0% in PAB > 20 µm), Bacteroidota (+ 17% in FLB and + 27% in PAB of 20–3 µm) and Fungi (+ 9.2% in pico-, + 6.1% in nano and + 3.7% in micro-protists) and a lower relative abundance of Cyanobacteria (− 22% in FLB, − 32% in PAB of 20–3 µm and -12% in PAB > 20 µm), Planctomycetota (− 6.1% in PAB of 20–3 µm and − 18% in PAB > 20 µm) and Chlorophyta (− 29% in FLB and − 13% in PAB of 20–3 µm) were observed.

The GR was also impacted, showing different dynamics compared to the DB. According to the model simulation (Fig. 5), the bay was impacted from Day 2 (Fig. 5B) and not from Day 1 as DB. However, salinity never showed values less than 34.4 (Fig. 5D). As a proxy of terrigenous inputs, the Si(OH)_4_ concentration (+ 13 µmol L^−1^) and HIX (+ 0.8) increased between days 2 and 3 (Additional file 2: Fig. S7). Microbial communities showed a double phase dynamic (Fig. 6). The first phase (Day 1–4) was marked by slow changes, as shown by a decrease in Dinoflagellata, especially Gymnodiniales in micro-protists dropping from 21% on Day 1 to 3.1% on Day 3. Conversely, Ochrophyta (+ 31% in nano- and + 39% in micro-protists between days 1 and 4) and Chlorophyta (+ 12% in pico-protists between days 1 and 3) increased. In particular, Bacillariophyta and Mamiellales explained by the rise of *Cyclotella* spp. (+ 11%) and *Ostreococcus* spp. (+ 12%) in the two groups. The second phase (days 4–6) showed a high increase in the proportion of unassigned Cercozoa in micro-protists (+ 29% between days 4 and 6) and a decrease in Chlorophyta in pico-protists (− 10%). In addition, an increase in heterotrophic taxa was observed during this second phase (+ 7.3% in pico-, + 4.5% in nano-, + 4% in micro-protists, + 11% in PAB > 20 µm between Days 4 and 6), with a decrease in photosynthetic taxa, such as Ochrophyta (− 37% in nano-, and − 22% in micro-protists) and Cyanobacteria (− 11% in PAB > 20 µm).

#### River plume impacts

The spatial impact of river runoff that followed Uesi extended beyond the coastal zone. Water mass drift was deducted from the trajectories of the two drifting buoys (Fig. 1C) and by modeling (Fig. 5). Changes within the water masses were observed from samples collected along trajectories of the buoys. A major variation was observed between Day 1 (in DB) and Day 2 for B1 and between Days 2 and 3 for B2. In 18 h, B1 drifted 18 km from the DB toward Boulari Bay (Fig. 1C). In B1, water temperature (+ 3 °C), salinity (+ 33), BIX (+ 0.5) and nano-protists, FLB and PAB of 20–3 µm richness (respectively + 226 ± 11, + 143 ± 63 and + 63 ± 25 ASVs) increased between Day 1 and 2. Conversely, a reduction in turbidity (-9.2 TSU), Si(OH)_4_ (-220 µmol L^−1^), HIX (− 13.5), POC (-1.5 mg L^−1^), Ni (− 213 µmol L^−1^) and pico- and micro-protist richness (− 91 ± 22 and − 121 ASVs) was registered. The water masses of both drifting buoys were characterized by lower abundances of heterotrophic taxa compared to offshore stations in dry campaigns in pico- (− 29% ± 3.0%), micro-protists (− 50% ± 3.3%) and FLB (− 10% ± 10%), whereas they showed an increase in PAB 20–3 µm (+ 16 ± 14%). The micro-protist community became clearly dominated by Cercozoa (> 50%) (Fig. 6), mostly due to 2 unassigned Cercozoa ASVs, ranging from 32 to 47% and from 18 to 28%, respectively. The composition of microbial communities differed from those found in offshore stations during the dry season. A larger proportion of Fungi [up to + 15% (B1-Day2)], Centroheliozoa (+ 2% ± 0.7% in pico-protists), and Planctomycetota [up to + 7% ± 0.3% (B1-Day2) in FLB] and a lower proportion of Radiolaria (− 5.4% ± 3%) and *Warnowia* spp. (− 5.4 ± 0.7%) were observed compared to dry season samples. The Flavobacteriaceae family showed a high proportion on B1 Day 2 (15%), and with the increased presence of some very rare genera (< 0.01%) in dry season offshore stations, such as *Flavobacterium* spp. (0.4% in B1-Day 2) and *Tenacibaculum* spp. (0.5%) (Fig. 7B).

## Discussion

This work especially highlighted the role of terrigenous inputs in shaping the coastal microbiome in a subtropical lagoon, both during steady (dry seasons) and exceptional conditions (rainy season). A clear dichotomy in ecological patterns between the two sampling conditions has been shown. We described microbiomes of the dry season, and we showed to what extent river runoff may alter typical conditions after a cyclone passage.

### Microbiome biodiversity and factors shaping its structuration

Coastal microbiome variations in space and time were explained here using a holistic approach based on the combination of chemical, biological, hydrological and meteorological parameters. Beyond classical parameters that structure marine microbial communities (nutrients, temperature and salinities), we considered metal concentrations (Ni, Cr, Co, Fe, Mn, Zn, Cu) and different OM parameters (POC, DON, a350, HIX, BIX, S1) known to influence microorganism diversity and physiology [99–102]. In particular, some OM proxies (a_350_, S_1_, BIX, HIX), rarely estimated in marine ecological surveys, in combination with particulate metal concentrations were chosen to characterize terrestrial inputs. We measured 21 environmental parameters in total, and their intercorrelations allowed us to finally select 8 major descriptors (temperature, salinity, Ni, POC, DON, S1, HIX and BIX) of the study area that allowed us to disentangle microbial community variability (Fig. 4). Those descriptors could be used for future ecological monitoring of the area. High metal concentrations in coastal waters are a peculiarity of New Caledonia ecosystems, which result from the ultramafic composition of the soil [41]. Metals of terrestrial rock origin arrive through soil erosion in coastal sites and influence microbial community diversity and composition. This cause‒effect mechanism is still barely studied. Although metals allow cell functioning, by several metalloproteins for example [103], some of them can be toxic in excess concentrations [104] and can alter physiological activities [105, 106].

#### Characteristics of dry season conditions

Although our dry season campaigns were carried out in different years (September 2019 and December 2020), both surveys were characterized by values of temperature, salinity, nutrients and Chl *a*, comparable to previous local studies [35, 44] and to other tropical and subtropical lagoon ecosystems [107–109]. This allowed us to consider that our dry season surveys well represent typical subtropical oligotrophic conditions. For the biodiversity composition, in both campaigns, the smallest microorganisms dominated in the systems (pico-protists showed 3 times more ASVs than micro-protists), as reported in other oligotrophic systems [5, 48]. Many studies on microbial biodiversity in similar environments have focused on pigmented microorganisms using microscopy, cytometry, fluorometry, and HCPLC-based surveys. They mainly showed a dominance of small cyanobacteria (*Synechococcus* and *Prochlorococcus*), diatoms and Chlorophyceae [110–112]. Our eDNA study, which also showed the dominance of Dinoflagellata, Ochrophyta and Chlorophyta, a biodiversity pattern already found in subtropical and tropical coastal ecosystems when eDNA was studied [113–115]. The non-negligible part of Haptophyta (mean relative abundance > 4% in both campaigns) and Cryptophyta (> 2%) in our data was only recorded by Gutiérrez-Rodriguez et al. 2021. Within the bacterial community, Proteobacteria, Actinobacteria, Bacteroidota and Cyanobacteria dominated, an expected pattern given the diversity and ecological importance of these groups in sea waters [116–120]. The difference in community between FLB (3–0.2 µm) and PAB (20–3 and > 20 µm) confirmed that those communities are distinct [121], with phyla more associated with OM, such as Bacteroidota, in PAB [122]. The lagoon of New Caledonia presents a high rate of N_2_ fixation [123], and cyanobacteria are considered important diazotrophic taxa. We found some diazotrophic cyanobacteria (UCYN-A, *Trichodesmium* sp., *Calothrix* group) in accordance with other studies [124, 125]. In addition, we identified Proteobacteria or Planctomycetes lineages that have the *nifH* gene, coding for the nitrogenase reductase needed to fix atmospheric nitrogen [126]. Overall, our study site showed a certain equilibrium between photoauto/mixotrophic and heterotrophic taxa in protist communities. Conversely, heterotrophic taxa dominated the bacterial communities, supporting the idea that microbial recycling is predominant in oligotrophic ecosystems [127, 128]. Especially for prokaryotes, future metagenomics and metatranscriptomic approaches will better describe and clarify the functional roles of the microbial community in this ecosystem.

#### Intraseasonal variability

Our eDNA observations provided new ecological information that allowed us to better describe subtropical coastal microbial compositions. We showed variability between September and December dry season microbiomes. This suggested a more complex microbial annual cycle than the usual microbial duality described at those latitudes between the dry and wet seasons [28, 129, 130]. In December, the augmentation of Dinoflagellata dominance in micro-protist, and the diminution of the proportion of photoautotrophic taxa in coastal areas could likely be explained by the higher temperature (+ 3.8 ± 0.7 °C) found in comparison to the September period. The slight increase in bacterial diversity (at the ASV level) associated with the decrease in DON concentration (− 1.4 ± 1.6 µmol L^−1^) could indicate a more active microbial loop in December. Given the influence of temperature on the microbial community [6], this factor could explain a significant part of the variability within the dry season before the arrival of frequent rains in the wet season. However, we recognize the limitations of our study regarding dry conditions, which is primarily based on two isolated snapshots of the area. A complete environmental eDNA-based annual cycle is needed to precisely describe and explain the microbial intraseasonality within the dry season, particularly to consider the interannual variability influenced by longer-scale climate phenomena such as ENSO.

#### Coastal spatial pattern

A spatial microbial biodiversity gradient was highlighted from the coast to the reef barrier, which was influenced by both terrigenous and oceanic inputs. Coastal-offshore gradients have already been reported in both temperate mid-latitude [131–133] and tropical coral reef ecosystems [134]. In our study, the Chl *a* concentrations and the number of ASVs of protists and bacteria increased close to the coast, consistent with what was described in Frade et al. (2020). This biodiversity gradient was associated with the quantity and quality of matter (Ni, POC, HIX, S1) (see also [135]) and the high dissolved metallic concentrations and bioavailability (not measured in this study) close to the coast [41, 43, 51]. The in-offshore gradient is shaped by hydrodynamics in many coral lagoon ecosystems. In typical conditions occurring during the dry season, the terrestrial impact is concentrated in bays and rapidly diluted offshore in the lagoon [44, 136]. Wind, tide and river flows are major factors shaping this gradient associated with the residence time of water bodies [137–139]. Waters with short residence times show less bacterial abundance [116], different microbial communities [140, 141], and less DOC [142], which corroborates the ecological patterns that we found offshore despite a nonhomogeneous and continuous sampling effort along this gradient.

In addition to these in-offshore gradients, our results demonstrated significant spatial heterogeneity between coastal stations. Some of them (A, D, G) were located at the mouth of rivers, where the natural features of the watersheds (size, vegetation, soil, etc.) influenced the microbiome composition of those sites. Other stations (F and E) were close to Nouméa city and then directly affected by urbanization, with the consequence that Chl *a* increased close to the city, as previously suggested [44, 143]. This increase was in accordance with the augmentation in the proportion of Dinoflagellata, Ochrophyta or Cyanobacteria that we found in our coastal stations. The high specificity of some coastal stations can be illustrated with the presence of some group only at a very local scale. As already shown [8, 144], the variability of coastal areas, shown by the heterogeneity of the environmental parameters, offers more ecological niches and thus richer microbial communities than in offshore areas. The different types of pressures occurring in our coastal stations directly influenced the microbial communities and the relative dominance of the taxonomic group by processes such as selection (e.g., by pollutant) or stimulation for growth (e.g., nitrate inputs).

### The multiscale impact of river runoff

Typical ecological patterns found during the dry season were disrupted by river runoff impact after the cyclone passage. Community structure perturbations can be described at both daily (one day after major freshwater input at DB-D1) and weekly time scales (during the 6 days of monitoring at DB and GR).

#### Daily perturbation

Drastic biological and hydrological changes occurred at the outlet of the Dumbéa River on the first day after runoff (Day 1 of the monitoring). Salinity dropped to 2, and the river was the vector of a huge quantity of dissolved and particulate components into the coastal area, including nutrients (NO_x_ (12.1 µmol L^−1^) and Si(OH)_4_ (226.7 µmol L^−1^)), OM (a350, HIX and BIX) and particulate metal (Ni 214 µg L^−1^) (Additional file 2: Fig. S7). At tropical and subtropical latitudes, rivers are the major source of nitrogen, phosphorus and silicate for coastal areas, mainly in the wet season [108, 145, 146]. Our situation represented an extreme condition considering that runoff input occurred after a cyclonic event. On Day 1 at DB, the massive input of particles resulted in an overall loss of diversity and a change in taxonomic and trophic microbial composition. The alpha diversity of communities dropped, mainly for pico-protists (− 50% of the ASV number). An increase in the relative abundance of heterotrophic taxa was observed in protist and PAB communities. Indeed, bacterial communities could respond quickly to the input of fresh OM, even at the daily time scale [147], as we observed.

New phyla and classes (Perkinsea in protists and Entotheonellaeota in bacteria) were reported after the cyclone but were not observed during the dry season campaigns. *S*ome riverine bacteria (Betaproteobacteria) were detected, especially members of Comamonadaceae, which are freshwater microorganisms [148, 149] and have already been recorded in the runoff waters of New Caledonia [150]. For the protists, the diatom genus *Cyclotella* typical of riverine or brackish water [151] increased in abundance. Some benthic taxa (e.g., diatoms) were also found after sediment resuspension caused by the river flow. The highly abundant ASV assigned to Ciliophora (Oxytrichidae family) observed at DB-D1 could also be a marker of freshwater or terrestrial input [152], but the limitation of our metabarcoding genetic assignation, based on generic (V4 18S rDNA) and not group-specific barcodes, does not allow us to certify it and to identify the species. Other taxa could be attributed to terrestrial organisms, such as some Methylomirabilota [153, 154], the bacterial strains GAL15 [155], and RCP2-54 [156]. Unassigned fungi, bacteria and protists found at DB on Day 1 could also be of soil origin. Certainly, the use of a reference database of soil ecosystems and/or database that take into account the biological endemism of the New Caledonia could improve the description of diversity.

#### Spatial extent of the perturbation

After the first day of river input, community and environmental variations were observed until the reef barrier, and followed different dynamics at DB and GR after the cyclone passage.

Post-cyclone meteorological conditions played an important role in influencing river plume spatial extension. After the Uesi cyclone, the river plume rapidly reached the coral barrier, 16 km from the Dumbéa River mouth, and was directed toward the south of the lagoon driven by an unusual wind direction (Figs. 1, 5). This hydrodynamic represented a specific case since trade winds usually transport water masses in a northwest direction [42]. Cyclones could have different consequences on marine ecosystems depending on local factors (e.g., geomorphology of the area) and meteorological conditions during and after the event, as well described by Devlin et al. [157]. The impact of the river plume on microbial communities is observed by the advection of taxa for the land/coast to offshore but also by the development and decrease of others as a consequence of the river inputs. Despite the dilution of the plume, some taxa typical of terrestrial or riverine areas were found along the buoy trajectories, such as Fungi (e.g., Exobasidiomycetes) or Betaproteobacteria (e.g., Comamonadaceae). The clear dominance of 2 unassigned Cercozoa is also unusual in the middle of the lagoon. However, members of this class have been reported as parasites of diatoms, and their presence might be related to the bloom observed [158]. Flavobacteriaceae, a member of Bacteroidota, also showed a higher relative abundance in the rainy season than in the dry season. This group is known to be a rapid member of OM degradation [159] and has already been considered an indicator of ecosystem degradation [130] and eutrophic condition [160]. Other microorganisms present in the dry season in middle and offshore stations decreased in importance after the river impact, such as the Dinoflagellate genus *Warnowia* or Radiolaria, which are both known as oceanic groups [5, 161–163]. Their decrease could be explained by biotic processes (competition, predation) or sensitivity to the unusual amount of particles in their environment.

#### Short-term time scale dynamics at coastal sites

The input of nutrients and OM by river runoff caused the development of photosynthetic organisms, as already observed in other river-influenced sites [29, 164–167]. In both DB and GR, the consequence of river runoff was an increase in the importance of diatom and cyanobacterial communities, and specific dynamics were highlighted for some groups (Fig. 7B), showing that various groups responded distinctly to cyclonic perturbation [168]. Diatoms (Bacillariophyta) picked 4 days after the cyclone, especially due to the contribution of *Cyclotella* spp. and then *Chaetoceros* spp. This result is in accordance with Cox *e*t al. 2006, who showed a peak of *Chaetoceros* spp. abundance and Chl *a* concentration 3–7 days after the river pulse impact. Regarding cyanobacteria, an increase in their relative abundance was observed in the PAB communities in both bays (Fig. 6B) and was quite short in time (visible only on Day 4 on DB and Day 3 on GR). Specific dynamics might depend on the affinity of the species for particulate matter, as demonstrated for PAB and FLB [44, 169]. Different kind of adaptation may explain the succession, with the temporary dominance of opportunistic and high-nutrient affinity species [170, 171] or biotic associations such as diatom-diazotroph assemblages [160, 167] in systems influenced by river impact.

Distinct community dynamics occurred at the local geographical scale between DB and GR. The DB was directly affected by the Dumbéa River, whereas the GR microbiome did not drastically vary in the first day after the cyclone but only 2–3 days after the river input, due to its geomorphology. Additionally, GR is known to be affected by industrial activities, notably with metallic contamination as high as in the mouth of rivers [143]. The higher residence time of water [139] combined with the potential pre-cyclone pollution can result in a quite complicated system at GR, which could explain the different dynamics between the two bays.

All our results showed that river runoff impacts in oligotrophic waters may have different impacts at weekly and even daily time scales and that at the local scale, geomorphological, hydrological and anthropogenic constraints may contribute to generating different dynamics and variable consequences on microbial communities.

### Ecosystem resilience

In the absence of pre-cyclone and long-term post-cyclone data that were hardly acquirable, we evaluated the ecosystem recovery by comparing post-cyclonic conditions with those encountered at two different periods of the dry season that we considered as baseline conditions of the studied environment. Despite some differences between the two different periods of the dry seasons, we found that post-cyclonic environmental conditions were very different than those of the dry season periods. We examined whether the post-cyclone biogeochemical and biological indicators in this study became similar to those of the dry season after 6 days of monitoring, and we speculated about how ecosystem resilience evaluation is dependent on the indicator considered.

#### Multiparameter analysis

The biological and biogeochemical variations during the 6 days after the cyclone showed an incomplete dynamic of the studied system at DB after the river impact. Despite the rapid return to usual salinity stability and hydrodynamic conditions, environmental descriptors representing both the metallic (Ni) and the organic compartments (terrigenous OM, Chl *a* concentration, community composition) showed quite different values after the river input in comparison with the dry season (Fig. 8). OM was not completely degraded after 6 days, as demonstrated by the high importance of functional groups of recycler microorganisms such as Bacteroidota, Desulfobacterota or Fungi [159, 172, 173]. Chl *a* picked after 6 days (2.5 µg L^−1^), suggesting that a bloom was still present and that the dynamics of photosynthetic groups were still in progress, corroborating other similar river-impacted areas where peaks of Chl *a* were observed after 2–10 days [136, 174, 175]. This result contrasted with the FLB community dynamics that remained quite homogeneous through space and time. This stability has already been recorded [176] and could support the size-plasticity hypothesis, stating that the smallest organisms could be less influenced by selection processes [177]. However, this homogeneity could be due to their dynamics being less linked to those of the particles than PAB communities. However, the microbial dynamics were assessed only with taxonomic information, and it could be relevant to analyze the recovery of the systems from a functional perspective, as taxonomic homogeneity may hide microbial functional variability [178].

#### Long-time scale perturbations

The studied river impact can be considered as a pulse disturbance, with a high intensity and a short duration [179]. This is characteristic of rainy events in the SW Pacific [136, 145]. Uesi was a magnitude 3 cyclone that impacted southern New Caledonia mainly by rain, mostly affecting the Dumbéa River basin (1188 m^3^ s^−1^ compared to the mean annual value of 3 m^3^ s^−1^) [53]. The direct impact of Uesi was similar to other study cases in both tropical [29, 111, 146, 180] and temperate areas [181]. However, Uesi was a medium-level cyclone, and stronger phenomena might have dramatic and longer-scale consequences in lagoon ecosystems. The combination of multiple events can result in cumulative effects that exert a more significant and enduring influence on coastal ecosystems [182]. This raises questions about the responses of the microbiome and ecosystem to repeated river inputs and the potential threats to coral reefs [180, 183]. At the scale of one or more wet seasons, regular monitoring could aid in evaluating the system resistance and resilience to multiple pulse disturbances, similar to Wijaya et al. [184].

## Conclusion

Using an oligotrophic, subtropical, river-impacted lagoon ecosystem as a study case, we showed that after an extreme disturbance, coastal to offshore gradients, intercoastal site variability, biological advection, and daily and local short-term community changes may occur in comparison to typical conditions observed during the dry season. At the same station impacted by river inputs and monitored for 6 days, we showed a recovery of the hydrodynamic and some physical parameters to typical values, but there were still some differences in biogeochemical and biological properties. These contrasting results suggest that when studying the resilience of an ecosystem, a multidisciplinary approach should be adopted since the recovery to typical conditions can vary according to the parameter considered. After an external perturbation, only holistic analyses of a studied ecosystem can provide a comprehensive evaluation of its resilience and the consequence of the perturbation. The implementation of longer multidisciplinary monitoring systems for the evaluation of the consequences of extreme events would allow us to study the dynamics of an impacted system and its resilience time over a long time, especially in our era of climate changes when extreme events might become more frequent and intense.

### Supplementary Information

**Additional file 1.** Additional methodological information with more details of the sampling site, laboratory procedures and functional classification of the communities. Supplementary figures showed electrophoresis gel of 16S and 18S marker gene for each campaign (Fig. S1–S6).

**Additional file 2.** Supporting information with the description of the ecological descriptors and the detailed spatial gradient illustrated by community composition. Table S1: Details of all sampling stations. Figure S1: Accumulation curves of 18S and 16S markers before the database cleaning. Figure S2: Meteorological conditions of the study site during 2019–2020 years. Figure S3: Correlation matrix of the 21 environmental parameters. Figure S4: nMDS on protist and bacterial communities. Figure S5: Spatial distribution of the taxonomic and functional composition of protist and bacterial communities during September 2019 campaign. Figure S6: Spatial distribution of the taxonomic and functional composition of protist and bacterial communities during December 2020 campaign. Figure S7: Variability over the three campaigns of the 8 key variables and other parameters.

**Additional file 3.** Dataset including all environmental parameters generated and analyzed in this study.

## Data Availability

Raw sequences are available at 10.12770/26840434-8354-4856-9d5e-e562c8de7252. Environmental data generated and analyzed during this study are included in this published article [see Additional file 3].
