# Supplementary material for: Subtropical coastal microbiome variations due to massive river runoff after a cyclonic event
Source: Environ Microbiome. 2024 Jan 30;19:10. doi: 10.1186/s40793-024-00554-9 (PMC10829310; doi:10.1186/s40793-024-00554-9)
Supplement: Supplementary file 1 — Additional file 1. Additional methodological information with more details of the sampling site, laboratory procedures and functional classification of the communities. Supplementary figures showed electrophoresis gel of 16S and 18S marker gene for each campaign (Fig. S1–S6). [file 40793_2024_554_MOESM1_ESM.docx]

Subtropical coastal microbiome variations due to massive river run-off after a cyclonic event

**Authors:**

M.Meyneng^1^, H. Lemonnier^2^, R. Le Gendre^2^, G. Plougoulen^2^, F. Antypas^2^, D. Ansquer^2^, J. Serghine^1^, S. Schmitt^1^, R. Siano^1^

^1^ IFREMER, DYNECO, BP70, Plouzané, France

^2^ IFREMER, LEAD, BP2059, 98846 Nouméa, New Caledonia

**Mail addresses:**

Corresponding authors: [raffaele.siano@ifremer.fr](mailto:raffaele.siano@ifremer.fr)

**Additional file 1**

**Heterogeneity of the sampling area and the anthropogenic pressures**

Watersheds are exposed to various land uses that result in different types of anthropogenic pressures on estuaries and adjacent bays. Dumbéa Bay (DB) is under urbanization pressure, and the terrigenous influences of the Dumbéa River are composed of five principal tributaries. Grande Rade Bay (GR) is impacted by urbanization and industrial activities, especially by the nickel metallurgic plant of the SLN (Société Le Nickel) industry. Despite the recent installation of sewage treatment systems, Sainte Marie Bay (SM) is still submitted to domestic wastewater runoff from Nouméa town, together with Coulée River runoff [1]. GR and SM bays are affected by eutrophication due to domestic effluents [2]. Boulari Bay receives inputs from the Coulée and Pirogue rivers. The Coulée River, composed of the upstream Coulée source and its main tributary named Lembi, delivers an important amount of lateritic material due to soil erosion impacted by mining [1, 3] and agricultural and urbanization activities. The Pirogue River is essentially impacted by the mining industry.

**Dissolved and particulate matter analyses**

For dissolved organic matter (DOM), water samples were filtered through 0.2 µm filters with Whatman® nucleopore filters. Absorbance spectra were measured from 250 to 700 nm in 10 cm pathlength quartz cells with a double beam Shimadzu UV-1800 spectrometer, with Milli-Q water as a blank. The cell was rinsed three times between samples with HCl at 10% and three times with demineralized water. Absorbance at 350 nm (A_350_) was measured and converted into the colored dissolved organic matter (CDOM) absorption coefficient (a_350_), a proxy of terrestrial input in water [4]. The quality of the OM was analyzed with the spectral slope derived from a_275_ and a_295_. The resulting slope (S1) indicated the origin, molecular weight and photochemical sensitivity of CDOM [5, 6]. High-resolution fluorescence spectroscopy was used to characterize the origin and composition of DOM [7]. The excitation emission matrices of fluorescence (FDOM) were determined using a Perkin Elmer LS55B from 200 to 500 nm and 280 to 550 nm for excitation and emission, respectively, using a 1 cm path length flow cell (more detailed in [4]). The index of humification (HIX) and the autotrophic productivity (BIX) were calculated with the fluorescence properties of CDOM [8, 9].

For particulate organic matter (POM) analyses, between 1 and 3 L of water samples were filtered through a 0.45 µm cellulose acetate Millipore filter (Merck Millipore, Billerca, MA, USA), depending on the turbidity of the water column. Extraction was performed by mineralization at 100 °C for 4 h after the addition of 6 mL ultrapure 37% HCl and 2 mL nitric acid. Particulate concentrations of nickel (Ni), cobalt (Co), manganese (Mn), iron (Fe), chromium (Cr), copper (Cu) and zinc (Zn) were measured by inductively coupled plasma spectrometry (ICP‒OES Varian 730-ES). The particulate organic carbon (POC) concentration was determined by filtering onto a combusted Whatman GF/F filter and after its decarbonatation with 37% fuming HCl on a SERCON integra 13C15N analyzer [10].

**Amplification methods**

For the V4 18S marker, the PCR mix recipe consisted of a total volume of 40 µl, including 0.5 µL 2x Phusion High-Fidelity DNA Polymerase (Thermo Scientific), 1 µL (10 mM) dNTP, 1.5 µL (3%) DMSO, 2 µL (10 μM) forward primer, 2 µl (10 µm) reverse primer, 31 µL sterile water and 2 µL of the filter-extracted eDNA sample. The PCR program consisted of an initial denaturation step at 98 °C for 30 s, followed by two sets of cycles (1) 12 × [98 °C (10 s), 53 °C (30 s), 72 °C (30 s)] and (2) 18 × [98 °C (10 s), 48 °C (30 s), 72 °C (30 s)].

For V4-V5 16S marker, the PCR mix consisted a total volume of 30 µL, with 0.12 µL Go Taq Flexi G2 (5 U/µL) (Promega), 6 µL Buffer Go Taq 5x, 1.8 µL MgCl2 (25 mM), 0.6 µL dNTP (10 mM), 0.45 forward primer (20 µM), 0.45 reverse primer (20 µM), 18.58 µL sterile water and 2 µL of the sample. The PCR program was an initial denaturation step at 95 °C for 5 min, followed by 32 cycles of [95 °C (30 s), 50 °C (60 s), 72 °C (60 s)].

For both markers, the last step was a final extension at 72 °C for 10 minutes.

**Functional classification**

In this study, we focused on the change between the heterotrophic and autotrophic regimes of the water system after the cyclonic event. Therefore, we voluntarily only separated autotrophic from heterotrophic taxa. For protists, taxa that own a plastid (either in a constitutive way or by having acquired it by mechanisms of endosymbiosis or kleptoplastid) were considered auto/mixo-trophic. Others were classified as heterotrophs since they cannot use light energy in any phase of their life cycle [10]. For bacteria, taxa able to perform photosynthesis (cyanobacteria and purple bacteria) were assigned to photoautotrophs, and all other bacterial phyla or classes were assigned to heterotrophs. ASVs assigned to an imprecise taxonomy (e.g., “Bacteria” or “Dinoflagellata”) were grouped as non-assigned in their respective group.

**References**

1. Fernandez J-M, Ouillon S, Chevillon C, Douillet P, Fichez R, Gendre RL. A combined modelling and geochemical study of the fate of terrigenous inputs from mixed natural and mining sources in a coral reef lagoon (New Caledonia). Mar Pollut Bull. 2006;52:320–31.

2. Fichez R, Chifflet S, Douillet P, Gérard P, Gutierrez F, Jouon A, et al. Biogeochemical typology and temporal variability of lagoon waters in a coral reef ecosystem subject to terrigeneous and anthropogenic inputs (New Caledonia). Mar Pollut Bull. 2010;61:309–22.

3. Hédouin L, Bustamante P, Churlaud C, Pringault O, Fichez R, Warnau M. Trends in concentrations of selected metalloid and metals in two bivalves from the coral reefs in the SW lagoon of New Caledonia. Ecotoxicol Environ Saf. 2009;72:372–81.

4. Dupouy C, Röttgers R, Tedetti M, Frouin R, Lantoine F, Rodier M, et al. Impact of Contrasted Weather Conditions on CDOM Absorption/Fluorescence and Biogeochemistry in the Eastern Lagoon of New Caledonia. Front Earth Sci. 2020;8:54.

5. Helms JR, Stubbins A, Ritchie JD, Minor EC, Kieber DJ, Mopper K. Absorption spectral slopes and slope ratios as indicators of molecular weight, source, and photobleaching of chromophoric dissolved organic matter. Limnol Oceanogr. 2008;53:955–69.

6. Hansen AM, Kraus TEC, Pellerin BA, Fleck JA, Downing BD, Bergamaschi BA. Optical properties of dissolved organic matter (DOM): Effects of biological and photolytic degradation. Limnol Oceanogr. 2016;61:1015–32.

7. Coble PG. Characterization of marine and terrestrial DOM in seawater using excitation-emission matrix spectroscopy. Mar Chem. 1996;51:325–46.

8. Huguet A, Vacher L, Relexans S, Saubusse S, Froidefond JM, Parlanti E. Properties of fluorescent dissolved organic matter in the Gironde Estuary. Org Geochem. 2009;40:706–19.

9. Zsolnay A, Baigar E, Jimenez M, Steinweg B, Saccomandi F. Differentiating with fluorescence spectroscopy the sources of dissolved organic matter in soils subjected to drying. Chemosphere. 1999;38:45–50.

10. Ramond P, Sourisseau M, Simon N, Romac S, Schmitt S, Rigaut-Jalabert F, et al. Coupling between taxonomic and functional diversity in protistan coastal communities. Environ Microbiol. 2019;21:730–49.


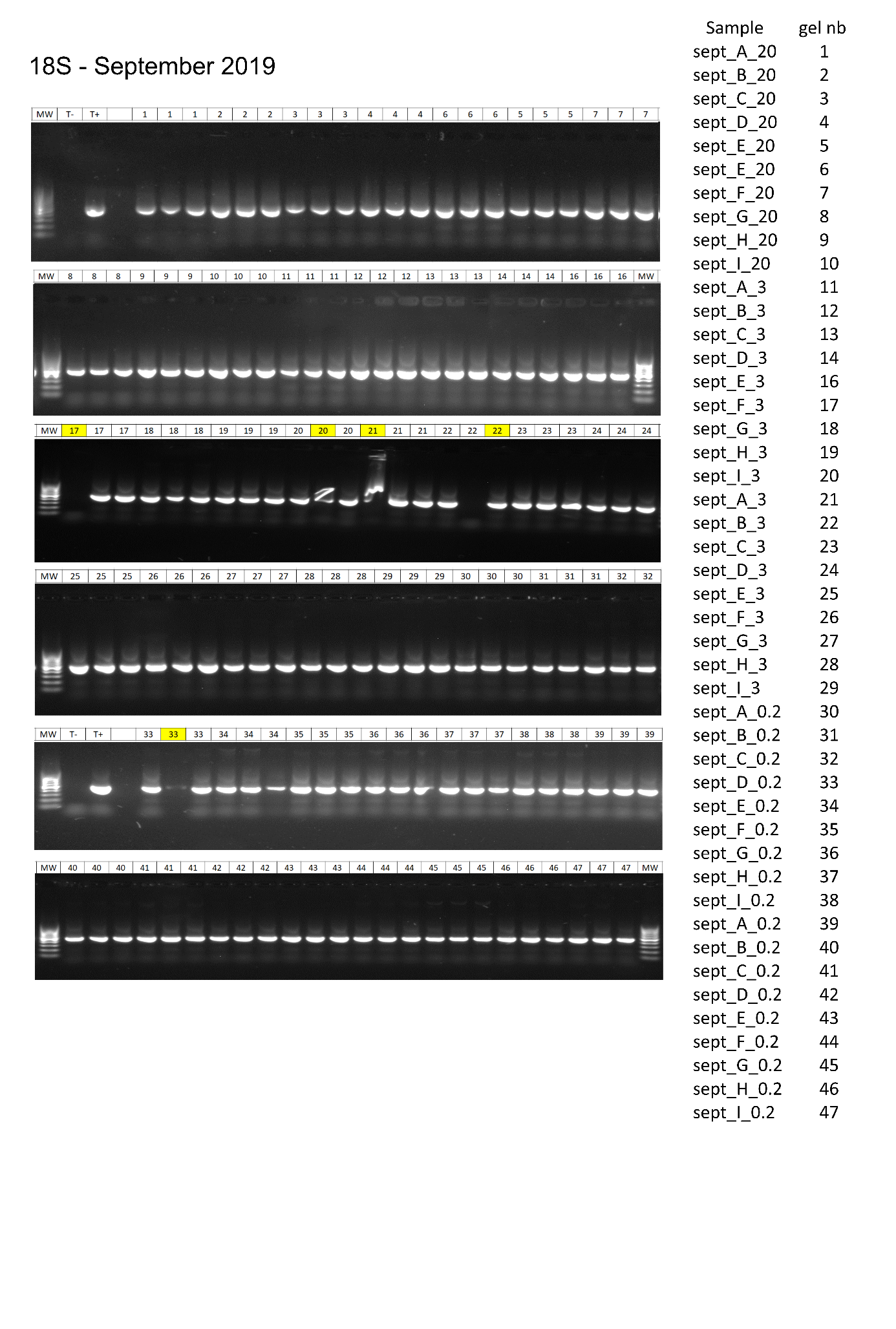


**Fig. S1**: Electrophoresis gel of 18S marker of the September 2019 campaign samples (T+: *Gambierdiscus* / T-: pure water). In each sample name the campaign, station and size fraction is precised (20: > 20µm; 3 = 20-3 µm; 02: 3-0.2 µm).


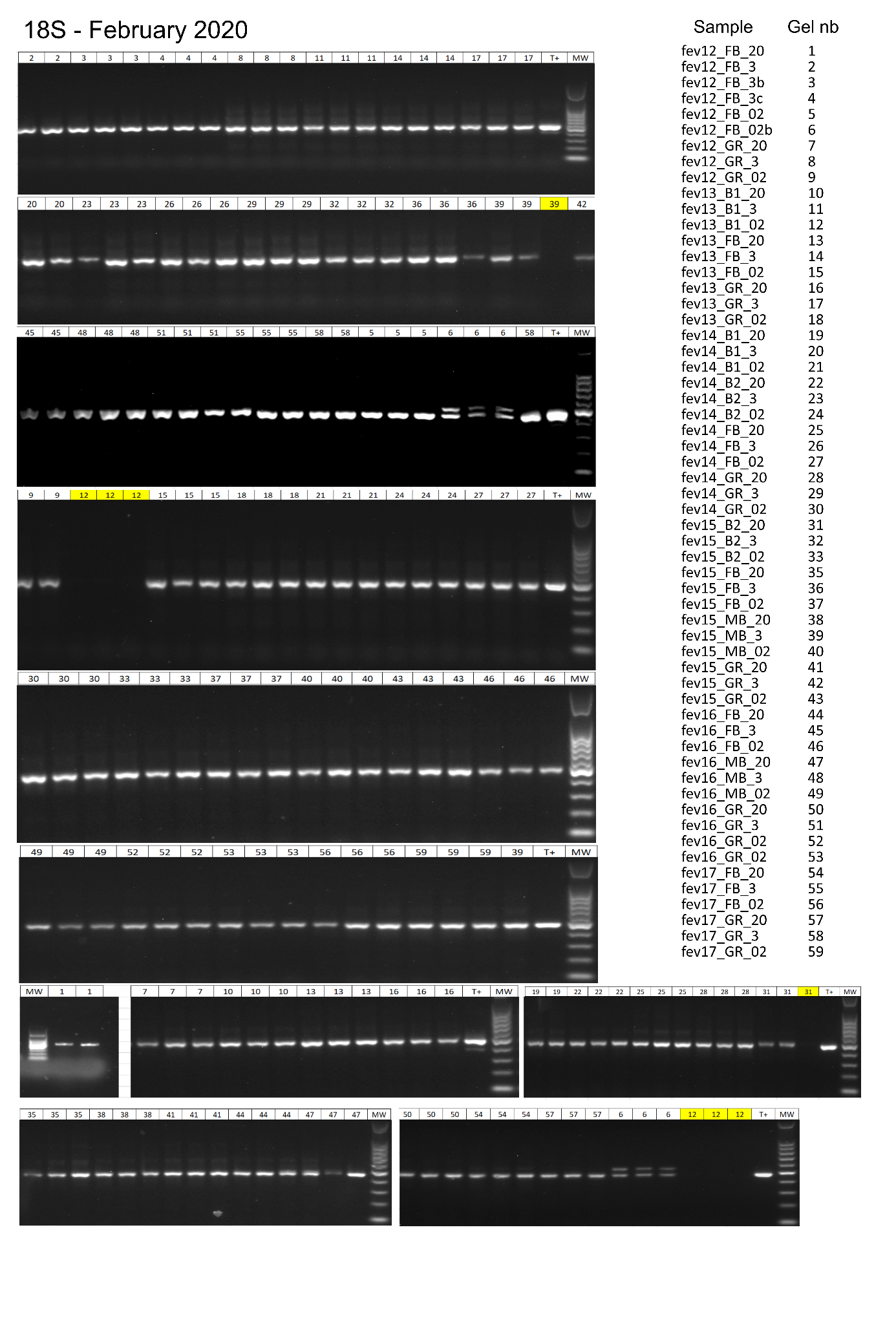


**Fig. S2**: Electrophoresis gel of 18S marker of the February 2020 campaign samples (T+: *Gambierdiscus*). In each sample name the campaign, station and size fraction is precised (20: > 20µm; 3 = 20-3 µm; 02: 3-0.2 µm).


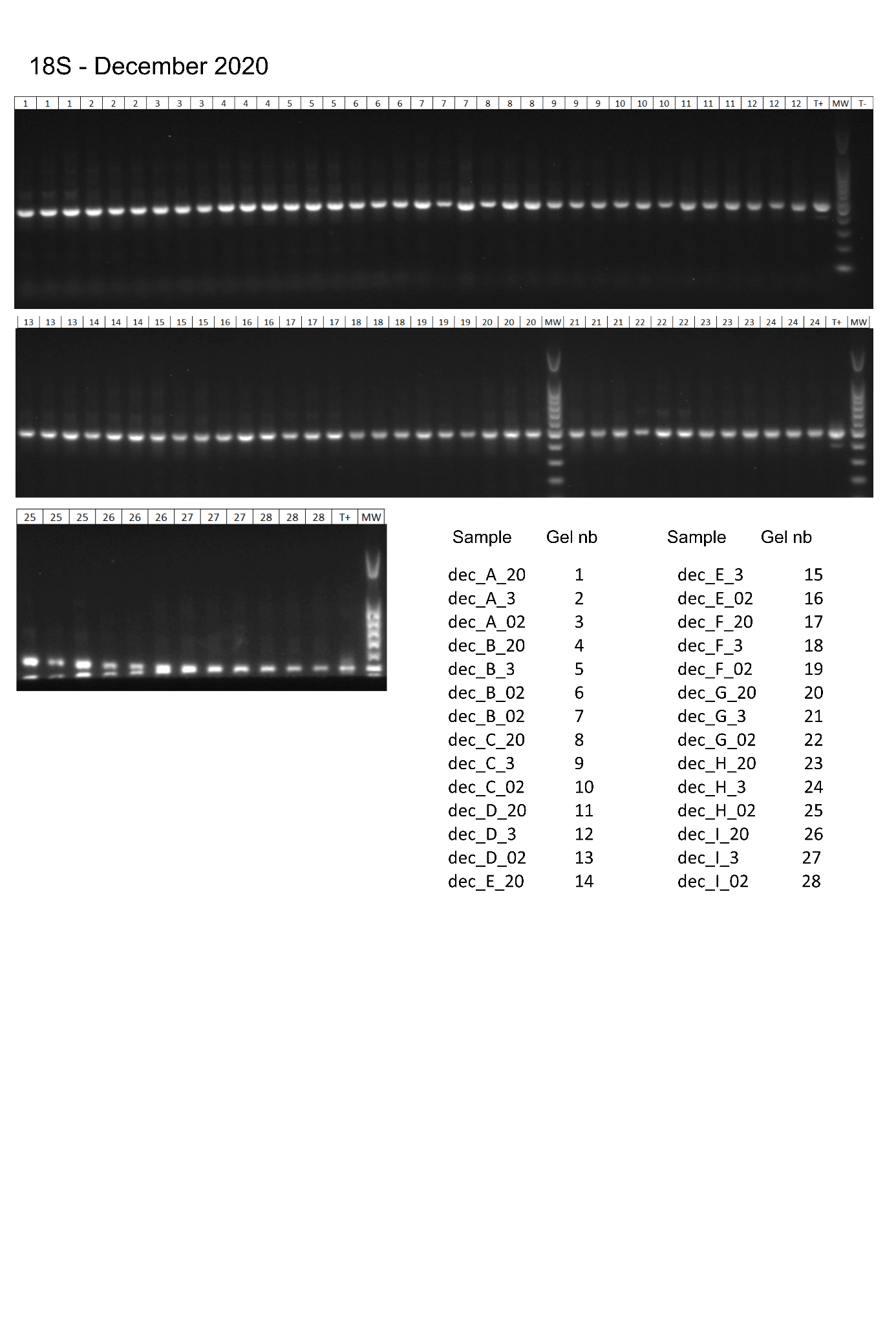


**Fig. S3**: Electrophoresis gel of 18S marker of the December 2020 campaign samples (T+: *Gambierdiscus* / T-: pure water). In each sample name the campaign, station and size fraction is precised (20: > 20µm; 3 = 20-3 µm; 02: 3-0.2 µm).


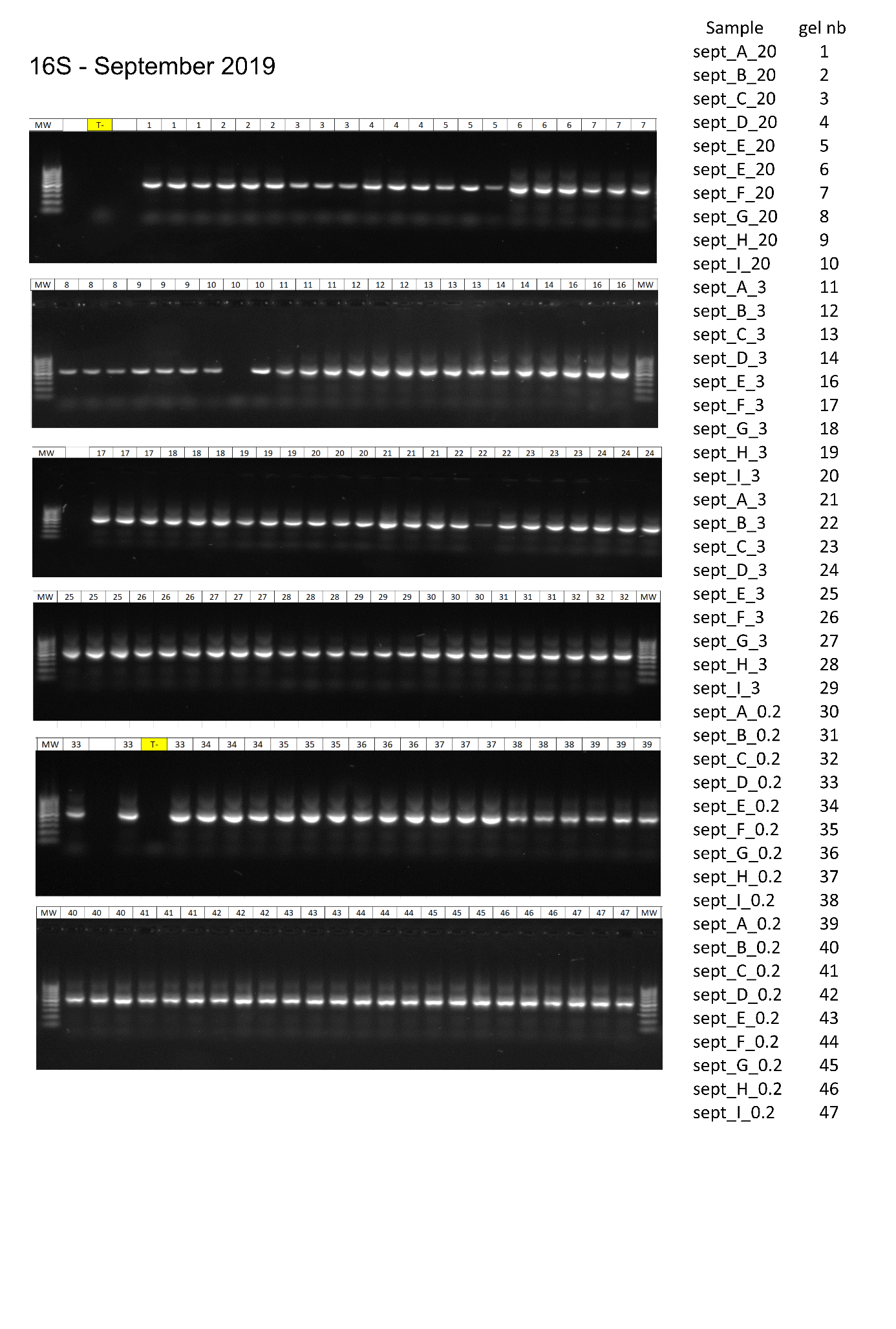


**Fig. S4**: Electrophoresis gel of 16S marker of the September 2019 campaign samples (T-: pure water). In each sample name the campaign, station and size fraction is precised (20: > 20µm; 3 = 20-3 µm; 02: 3-0.2 µm).


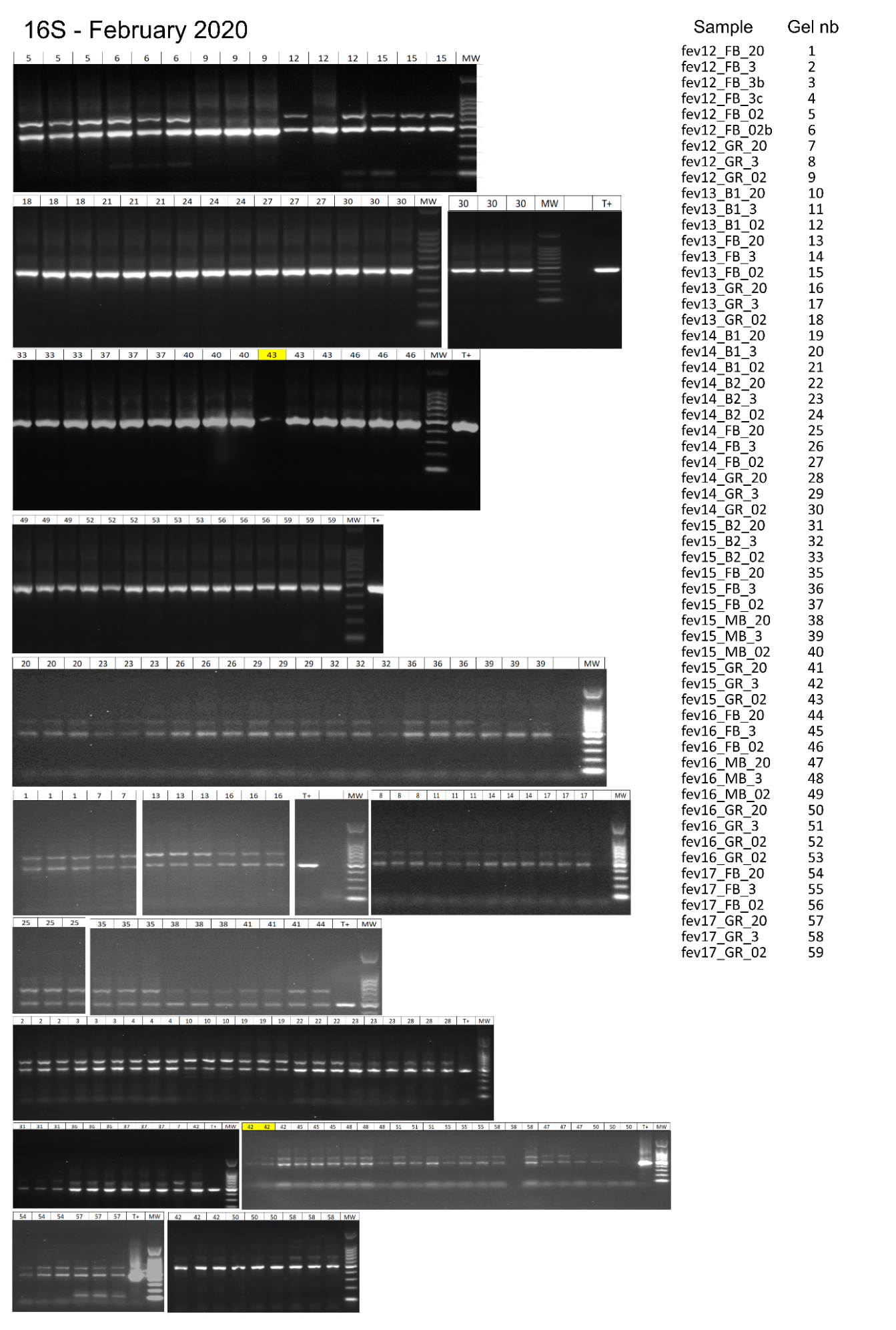
 **Fig. S5:** Electrophoresis gel of 16S marker of the February 2020 campaign samples (T+: *Vibrio* sp. / **T-:** pure water). In each sample name the campaign, station and size fraction is precised (20: > 20µm; 3 = 20-3 µm; 02: 3-0.2 µm).


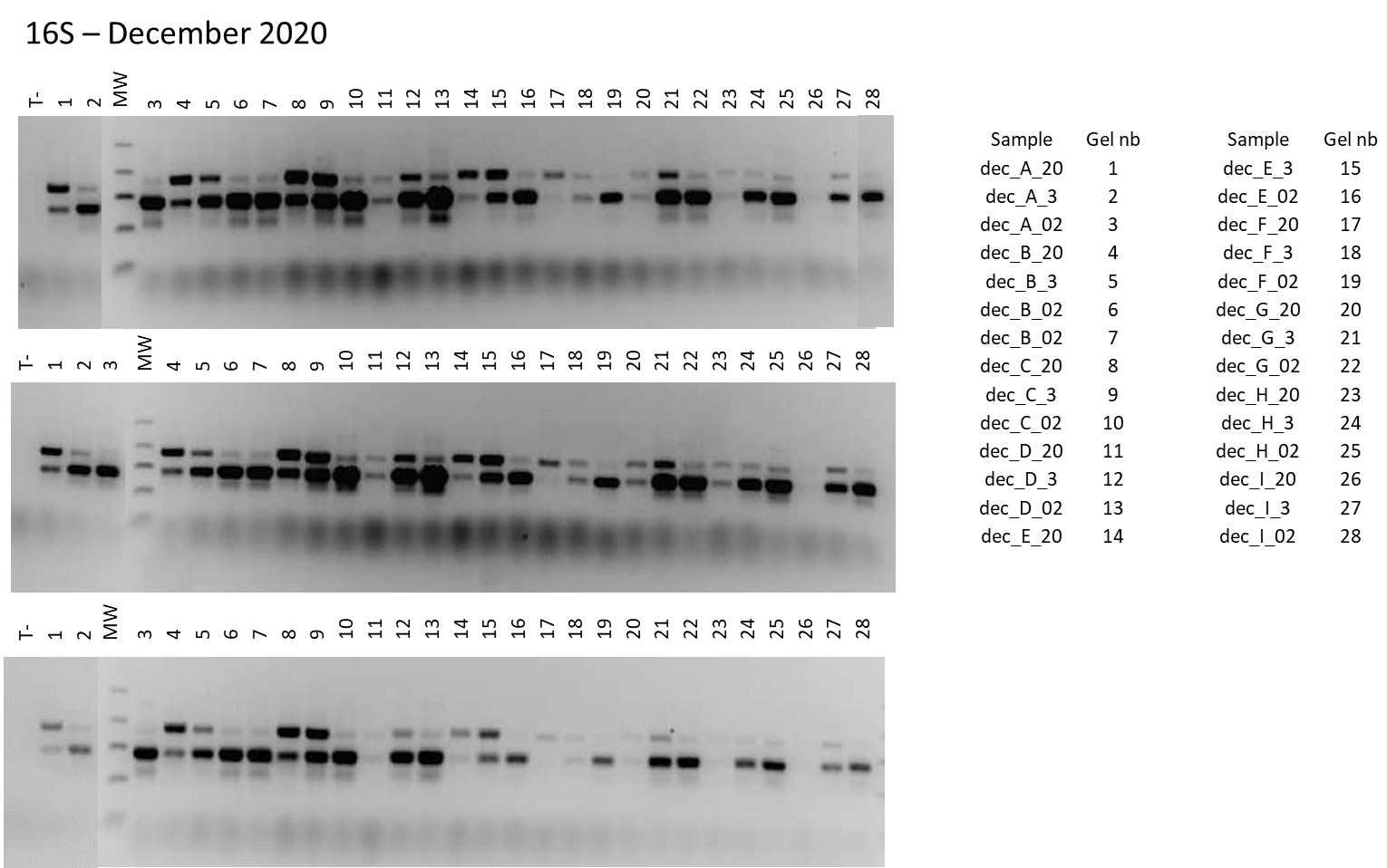


**Fig. S6**: Electrophoresis gel of 16S marker of the December 2020 (1st replicate) campaign samples (T-: pure water). In each sample name the campaign, station and size fraction is precised (20: > 20µm; 3 = 20-3 µm; 02: 3-0.2 µm).
