# Supplementary material for: Subtropical coastal microbiome variations due to massive river runoff after a cyclonic event
Source: Environ Microbiome. 2024 Jan 30;19:10. doi: 10.1186/s40793-024-00554-9 (PMC10829310; doi:10.1186/s40793-024-00554-9)
Supplement: Supplementary file 2 — Additional file 2. Supporting information with the description of the ecological descriptors and the detailed spatial gradient illustrated by community composition. Table S1: Details of all sampling stations. Figure S1: Accumulation curves of 18S and 16S markers before the database cleaning. Figure S2: Meteorological conditions of the study site during 2019–2020 years. Figure S3: Correlation matrix of the 21 environmental parameters. Figure S4: nMDS on protist and bacterial communities. Figure S5: Spatial distribution of the taxonomic and functional composition of protist and bacterial communities during September 2019 campaign. Figure S6: Spatial distribution of the taxonomic and functional composition of protist and bacterial communities during December 2020 campaign. Figure S7: Variability over the three campaigns of the 8 key variables and other parameters. [file 40793_2024_554_MOESM2_ESM.docx]

Subtropical coastal microbiome variations due to massive river run-off after a cyclonic event

**Authors:**

M.Meyneng^1^, H. Lemonnier^2^, R. Le Gendre^2^, G. Plougoulen^2^, F. Antypas^2^, D. Ansquer^2^, J. Serghine^1^, S. Schmitt^1^, R. Siano^1^

^1^ IFREMER, DYNECO, BP70, Plouzané, France

^2^ IFREMER, LEAD, BP2059, 98846 Nouméa, New Caledonia

**Mail addresses:**

Corresponding authors: [raffaele.siano@ifremer.fr](mailto:raffaele.siano@ifremer.fr)

**Additional file 2**

**Choice of ecological descriptors**

The whole dataset of the 21 measured environmental parameters are available in Additional file 3. Some of them were significantly correlated with each other in all datasets as shown in Fig. S3 (see below). Eight parameters were chosen as key descriptors. Salinity and water temperature were chosen to characterize the hydrology of the sampled waters. The particulate compartment was represented by POC (particulate organic carbon), an indicator of the quantity of POM and Ni (particulate nickel) concentrations. Ni was positively correlated with the majority of other particulate metal concentrations (Spearman coefficient of 0.99 with chrome, 0.96 with iron, 0.95 with manganese and 0.84 with cobalt); therefore, it was chosen to represent metal concentrations in the water column. The DOM was characterized by S1 (Spearman coefficient of -0.87 with a350), in addition to HIX (Humification Index), BIX (Biological Index) and DON (Dissolved Organic Nitrogen).

**Spatial gradient illustrated by community composition**

The spatial gradient visible in several environmental parameters was also illustrated by the community composition. The proportion of Cercozoa was higher in coastal stations, with 6.2 ± 5.5% for pico-, 3.6 ± 4.7% for nano- and 1.5 ± 2.3% for micro-protist communities against 2.4 ± 0.9%, 0.9 ± 0.5% and 0.5 ± 0.9% on average in middle and offshore stations. The same pattern was observed for Ochrophyta with nano- and micro-protists communities, which were 23 ± 19% and 18 ± 9.3% at the coastal stations and 7.7 ± 3.3% and 7.5 ± 6.45% at the middle and offshore stations, respectively. In contrast, Radiolaria were more dominant at middle and offshore stations, with 9.8 ± 5.6% for pico-, 2.4 ± 4.2% for nano- and 3.8 ± 2.9% for micro-protist communities against 1.2 ± 1.3%, 0.4 ± 0.4% and 1.6 ± 1.6% in coastal stations, respectively. This gradient was also visible for some bacterial phyla, as Planctomycetota that showed higher relative abundance in middle and offshore stations in 20-3 µm PAB with 14.4 ± 8.6% against only 5.9 ± 4.0% in coastal stations. Photoautotrophs decreased to the offshore stations with -24 ± 20% on average for pico- (except for the Pirogues transect in December) and -30 ± 21% for micro-protists.

**Supplementary figures and table**

| Season | Station | Site | Coordinates | Date | Hour |
| --- | --- | --- | --- | --- | --- |
| Dry season | A | Dumbéa bay | 22°12,436S 166°23,635E | 09/23/2019 | 10:37 |
|  |  |  |  | 12/08/2020 | 8:20 |
|  | B | Moïse station | 22°15,808S 166°19,585E | 09/23/2019 | 10:07 |
|  |  |  |  | 12/08/2020 | 9:05 |
|  | C | Dumbéa pass | 22°19,373S 166°17,760E | 09/23/2019 | 9:30 |
|  |  |  |  | 12/08/2020 | 9:35 |
|  | D | Coulée bay | 22°15,297S 166°32,724E | 09/24/2019 | 10:09 |
|  |  |  |  | 12/09/2020 | 8:15 |
|  | E | Sainte Marie bay | 22°17,162S 166°27,780E | 09/24/2019 | 10:39 |
|  |  |  |  | 12/09/2020 | 8:50 |
|  | F | Grande Rade | 22°15,299S 166°25,494E | 09/24/2019 | 11:13 |
|  |  |  |  | 12/09/2020 | 9:25 |
|  | G | Pirogue bay | 22°18,834S 166°40,660E | 09/25/2019 | 8:28 |
|  |  |  |  | 12/10/2020 | 8:00 |
|  | H | Lagoon | 22°24,035S 166°34,913E | 09/25/2019 | 9:02 |
|  |  |  |  | 12/10/2020 | 8:25 |
|  | I | Boulari pass | 22°27,342S 166°31,150E | 09/25/2019 | 9:28 |
|  |  |  |  | 12/10/2020 | 9:00 |
| Post cyclone | DB | Dumbéa Bay | 22°19,554S 166°41,583E | 02/12/2020 | 14:39 |
|  |  |  |  | 02/13/2020 | 9:33 |
|  |  |  |  | 02/14/2020 | 9:50 |
|  |  |  |  | 02/15/2020 | 8:47 |
|  |  |  |  | 02/16/2020 | 8:30 |
|  |  |  |  | 02/17/2020 | 7:48 |
|  | GR | Grande Rade | 22°24'661S 166°37'593E | 02/12/2020 | 14:54 |
|  |  |  |  | 02/13/2020 | 10:05 |
|  |  |  |  | 02/14/2020 | 10:07 |
|  |  |  |  | 02/15/2020 | 9:15 |
|  |  |  |  | 02/16/2020 | 8:55 |
|  |  |  |  | 02/17/2020 | 8:03 |
|  | B1-D2 | Buoy 1 trajectory | 22°21,157S 166°22,329E | 02/13/2020 | 8:37 |
|  | B1-D3 |  | 22°19,203S 166°32,398E | 02/14/2020 | 8:34 |
|  | B2-D3 | Buoy 2 trajectory | 22°17,259S 166°20,429E | 02/14/2020 | 9:20 |
|  | B2-D4 |  | 22°19,157S 166°19,176E | 02/15/2020 | 8:00 |

**Table S1:** Details of all sampling stations.

**
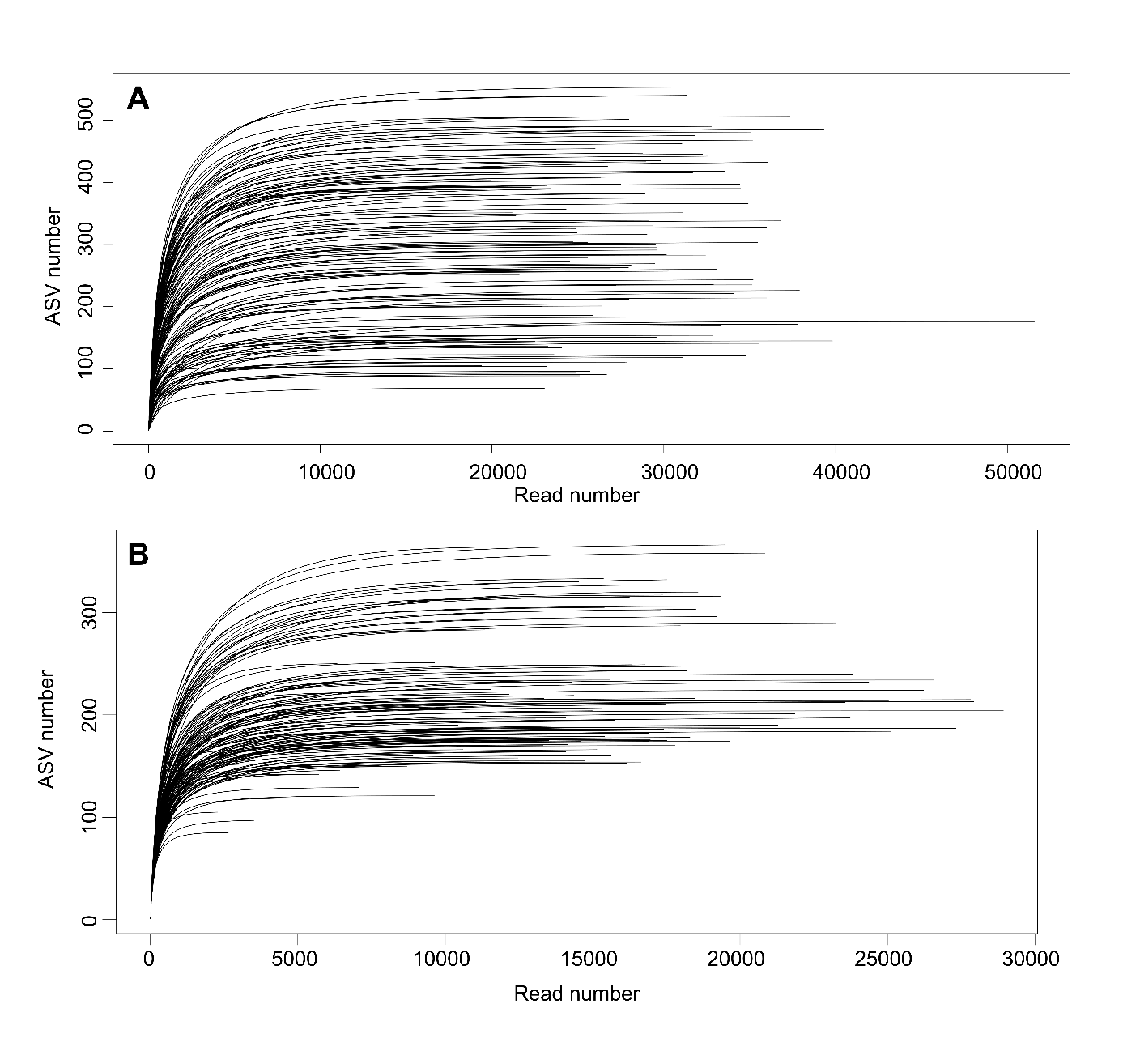
**

**Fig. S1:** Accumulation curves of (A) 18S and (B) 16S markers before the database cleaning, representing the number of ASV depending on the read number of each sample.

**
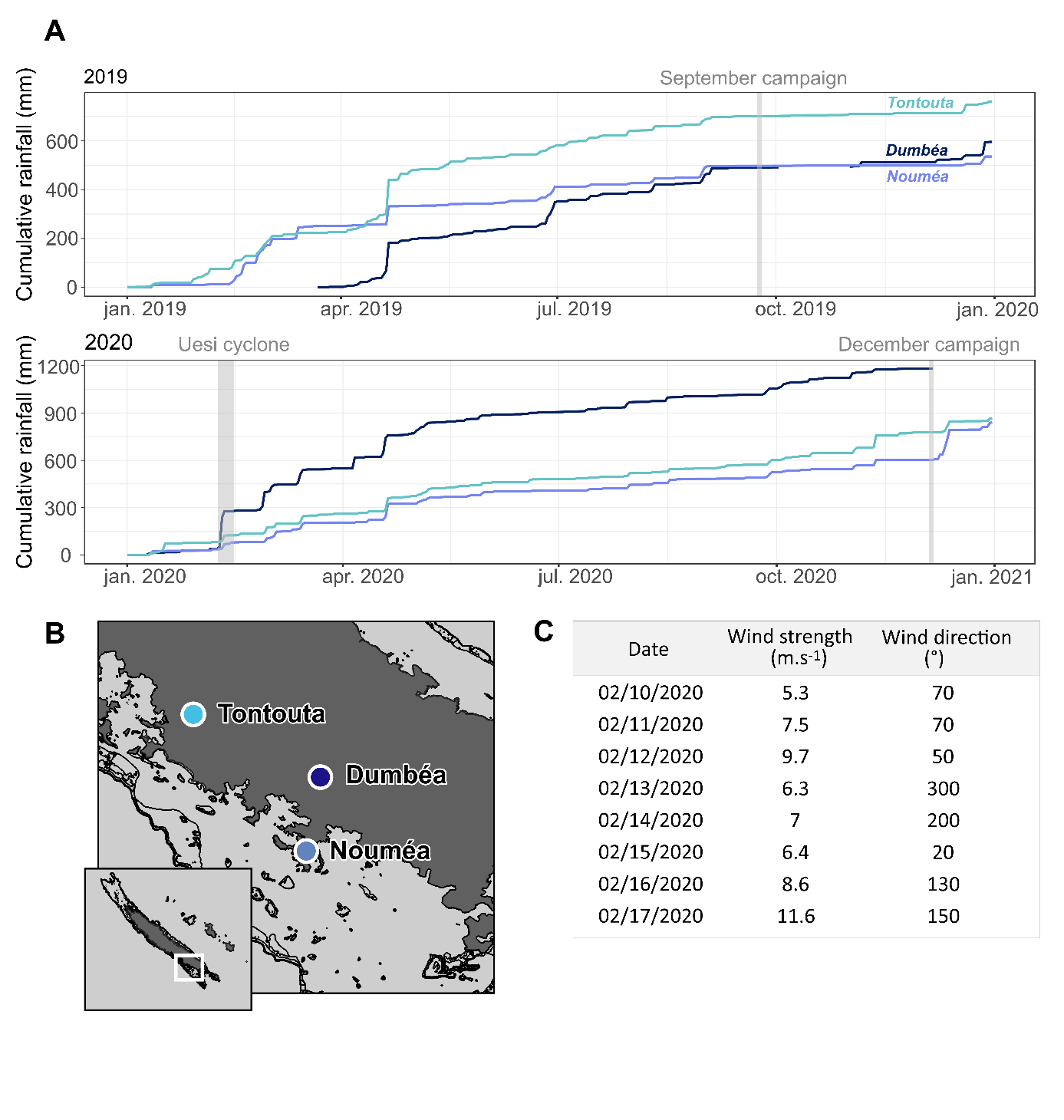
Fig. S2:** Meteorological conditions with (A) cumulative rain on year 2019 and 2020, (B) localization of fixed stations close to the study site and (C) wind regime during and after Uesi cyclone on the Nouméa station.

**
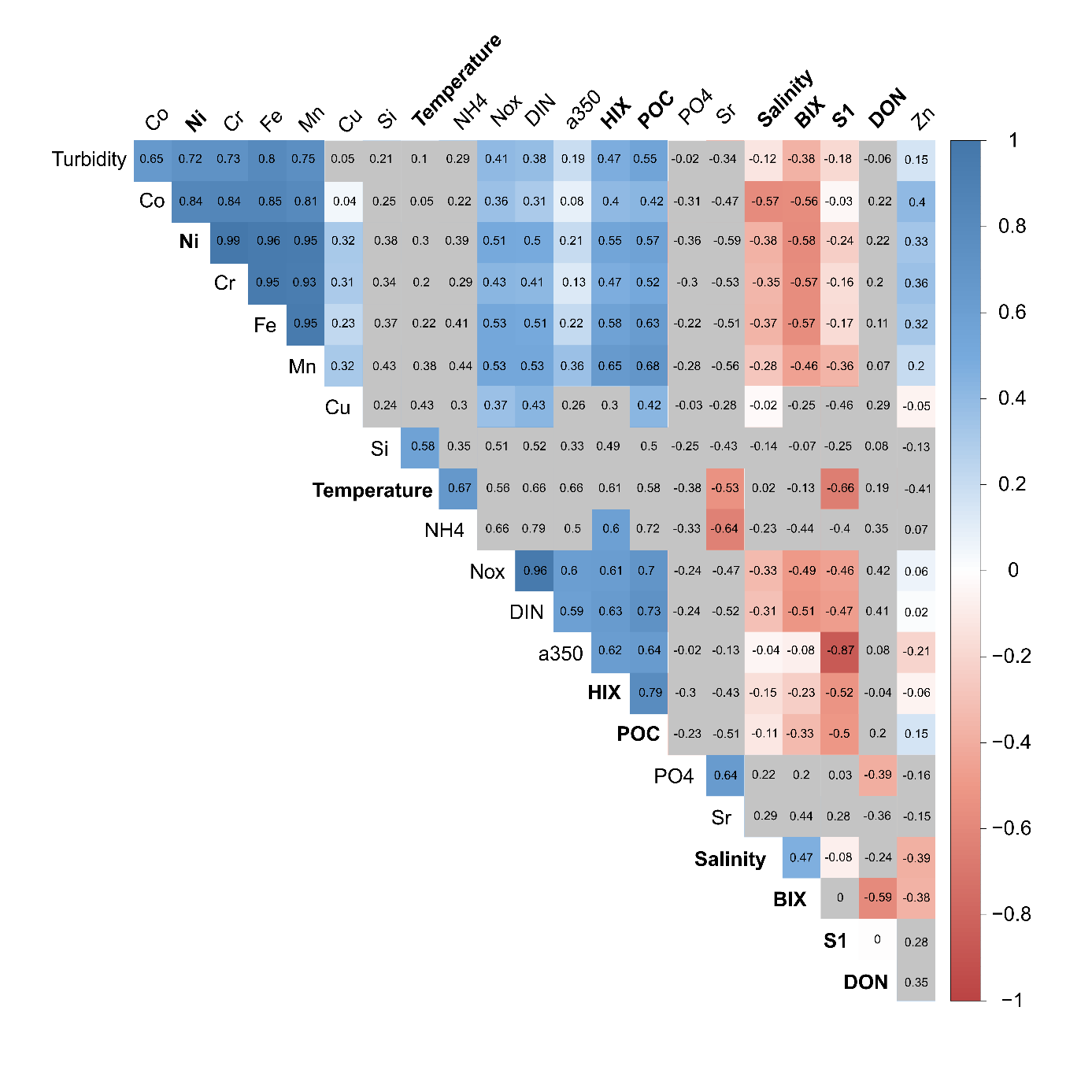
**

**Fig. S3:** Correlation matrix of 21 environmental variables, based on spearman coefficient (grey boxes represent non-significate correlations, with p-value > 0.05). The eight chosen descriptors are specified in bold.

**
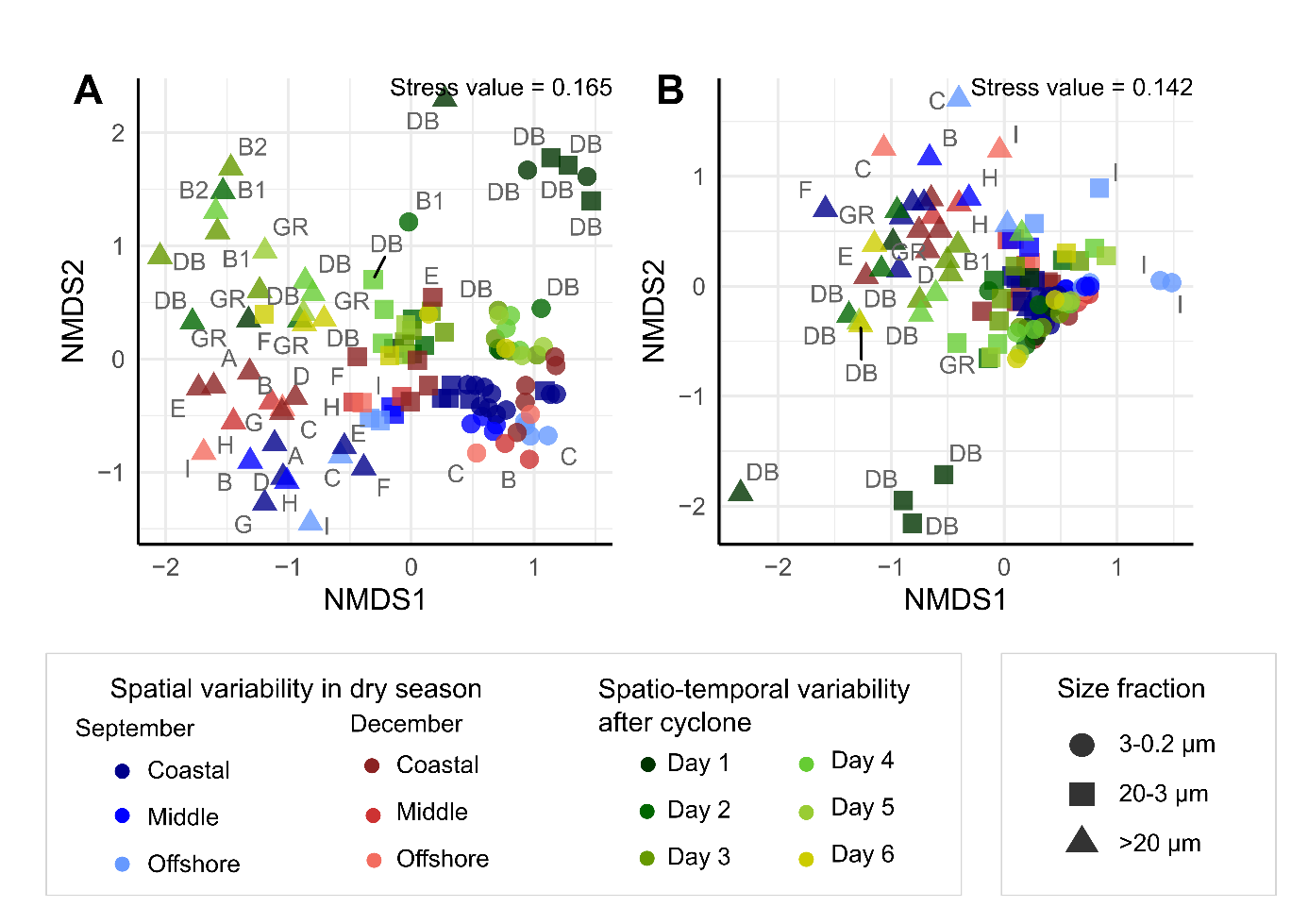
**

**Fig. S4**: Non-metric dimensional scaling (nMDS) performed on Bray-Curtis dissimilarities of (A) protists and (B) bacterial communities of all samples.


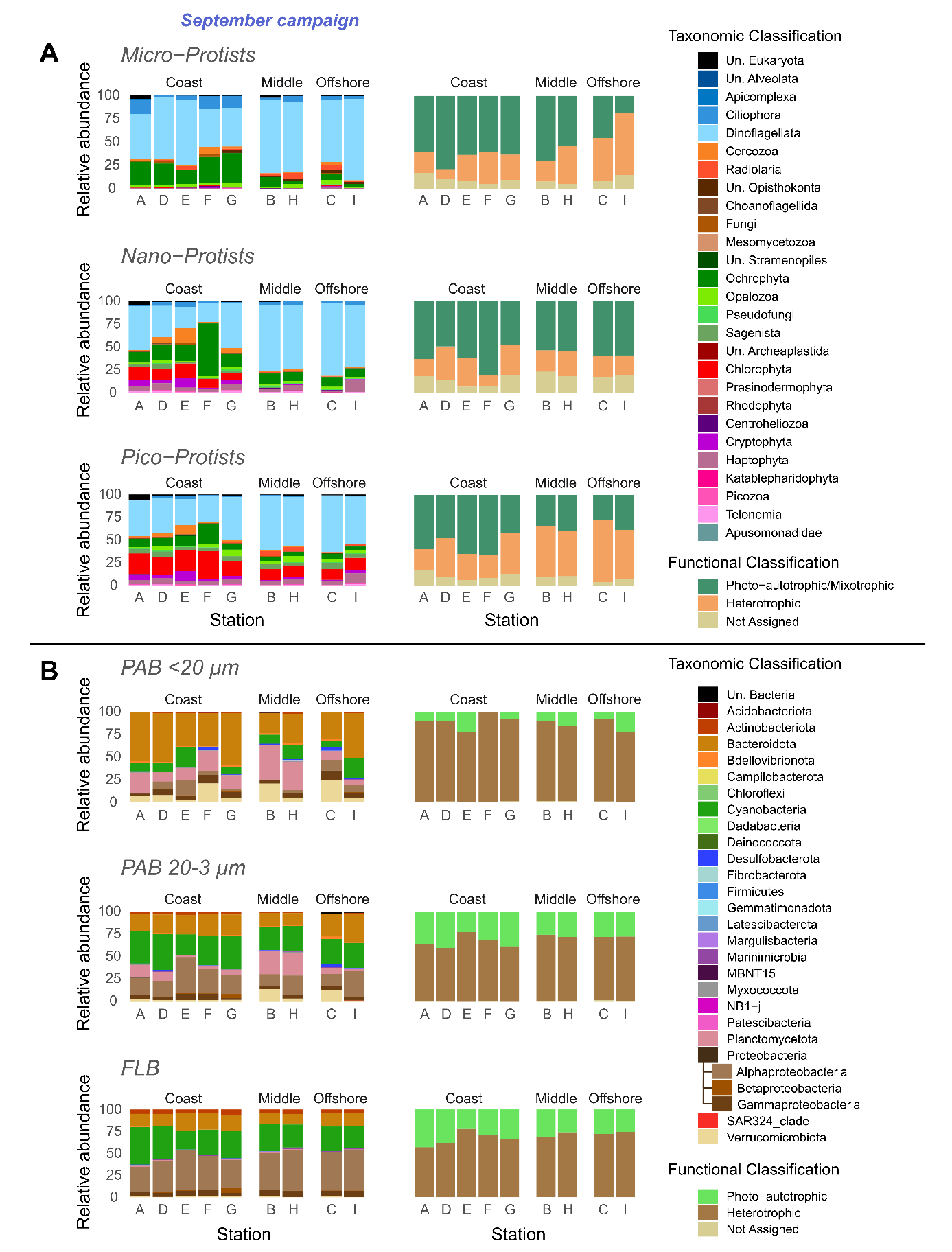


**Fig. S5:** Spatial distribution of the taxonomic and functional composition of protists (A) and bacterial (B) communities, in relative abundance (%), during September 2019 campaign. (Unassigned: Un.)


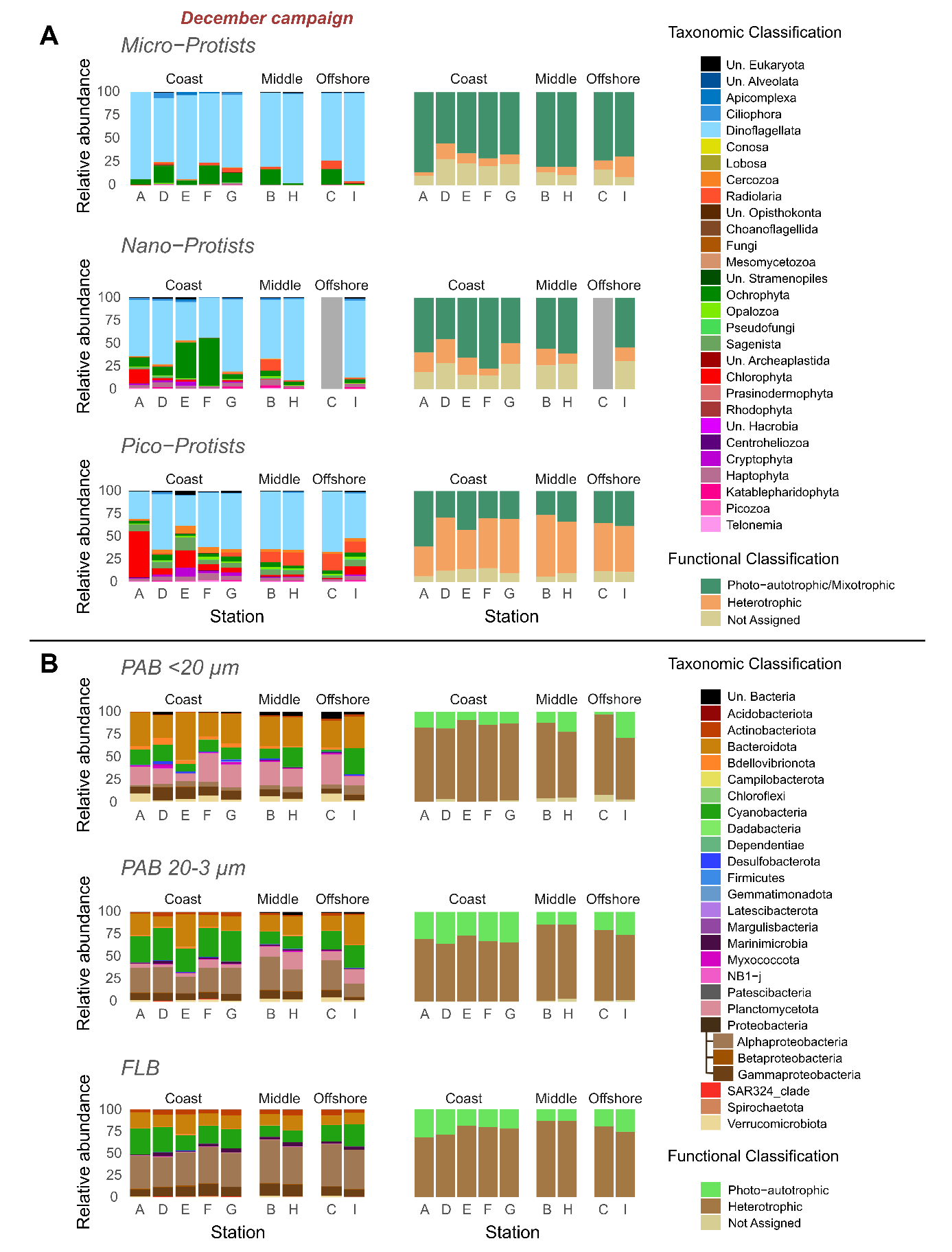


**Fig. S6:** Spatial distribution of the taxonomic and functional composition of protists (A) and bacterial (B) communities, in relative abundance (%), during December 2020 campaign. (Unassigned: Un.)

**
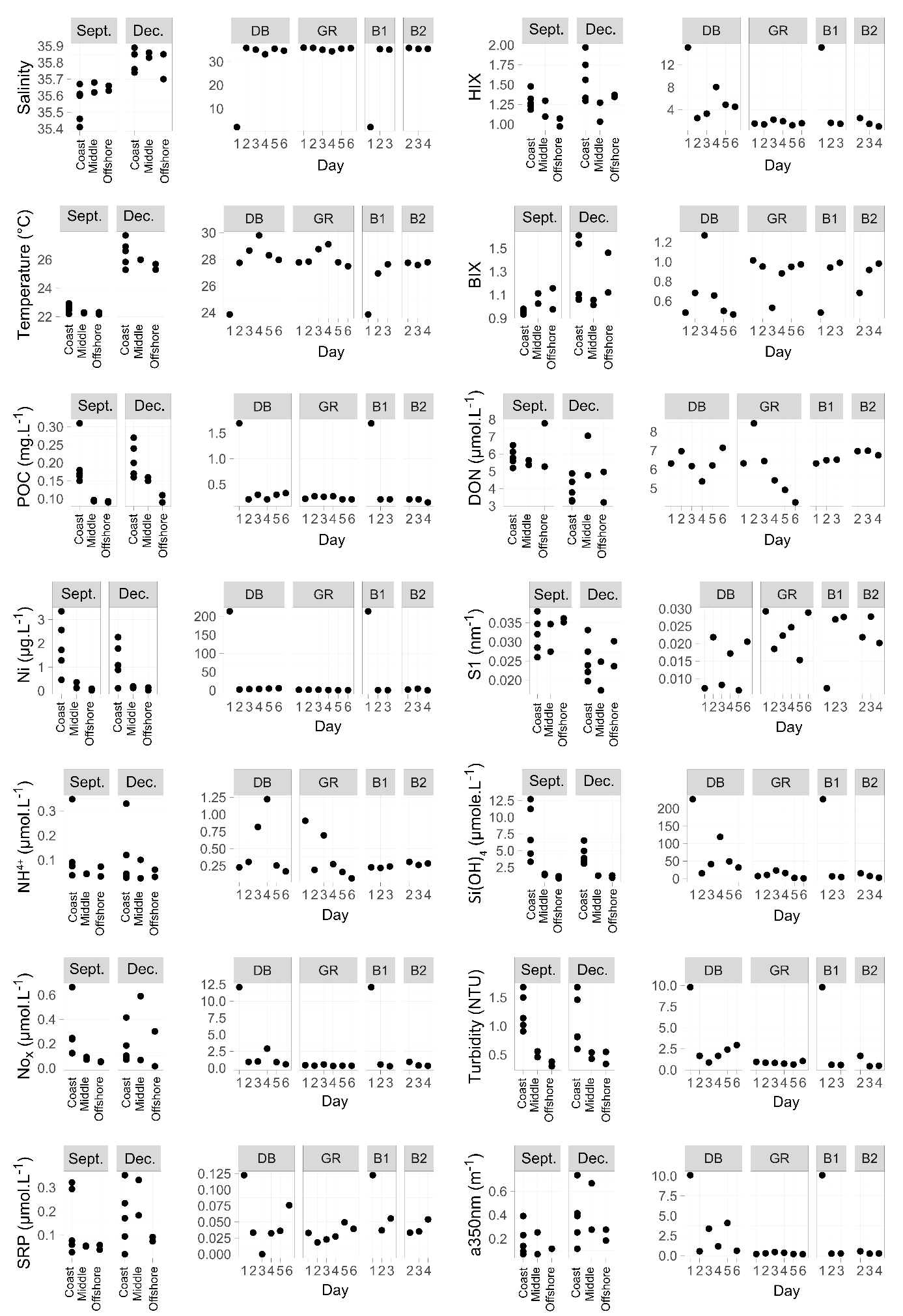
Fig. S7:** Variability over the three campaigns of the 8 key variables and other parameters
